# An assessment of true and false positive detection rates of stepwise epistatic model selection as a function of sample size and number of markers

**DOI:** 10.1038/s41437-018-0162-2

**Published:** 2018-11-15

**Authors:** Angela H. Chen, Weihao Ge, William Metcalf, Eric Jakobsson, Liudmila Sergeevna Mainzer, Alexander E. Lipka

**Affiliations:** 1 Department of Statistics, University of Illinois at Urbana-Champaign, Urbana, IL 61801 USA; 2 Beckman Institute for Advanced Science and Technology, University of Illinois at Urbana-Champaign, Urbana, IL 61801 USA; 3 Center for Biophysics and Computational Biology, University of Illinois at Urbana-Champaign, Urbana, IL 61801 USA; 4 Department of Computer Sciences, Rose-Hulman Institute of Technology, Terre Haute, IN 47803 USA; 5 Carl R. Woese Institute for Genomic Biology, University of Illinois at Urbana-Champaign, Urbana, IL 61801 USA; 6 Neuroscience Program, University of Illinois at Urbana-Champaign, Urbana, IL 61801 USA; 7 National Center for Supercomputing Applications, University of Illinois at Urbana-Champaign, Urbana, IL 61801 USA; 8 Department of Molecular and Integrative Physiology, University of Illinois at Urbana-Champaign, Urbana, IL 61801 USA; 9 Department of Crop Sciences, University of Illinois at Urbana-Champaign, Urbana, IL 61801 USA

**Keywords:** Genome-wide association studies, Quantitative trait

## Abstract

Association studies have been successful at identifying genomic regions associated with important traits, but routinely employ models that only consider the additive contribution of an individual marker. Because quantitative trait variability typically arises from multiple additive and non-additive sources, utilization of statistical approaches that include main and two-way interaction marker effects of several loci in one model could lead to unprecedented characterization of these sources. Here we examine the ability of one such approach, called the Stepwise Procedure for constructing an Additive and Epistatic Multi-Locus model (SPAEML), to detect additive and epistatic signals simulated using maize and human marker data. Our results revealed that SPAEML was capable of detecting quantitative trait nucleotides (QTNs) at sample sizes as low as *n* = 300 and consistently specifying signals as additive and epistatic for larger sizes. Sample size and minor allele frequency had a major influence on SPAEML’s ability to distinguish between additive and epistatic signals, while the number of markers tested did not. We conclude that SPAEML is a useful approach for providing further elucidation of the additive and epistatic sources contributing to trait variability when applied to a small subset of genome-wide markers located within specific genomic regions identified using a priori analyses.

## Introduction

The ability to identify genomic regions containing gene(s) associated with quantitative phenotypes has great potential for elucidating the genetic architecture of traits (e.g., number of genes, their effect sizes, additive vs. non-additive sources), as well as identifying targets for marker-assisted selection in plants and animals and therapy in humans. One analysis that seeks to identify such regions is the genome-wide association study (GWAS), in which statistical analyses are conducted on a set of markers spanning a species’ entire genome to determine which marker subsets exhibit the strongest associations with a trait of interest (reviewed in Lipka et al. 2015). In general, statistically significant marker-trait associations suggest that functional variants for the trait under study are located in the surrounding genomic region. To date, GWAS has been able to identify genes associated with many important traits, e.g., predisposition to breast cancer and diabetes in humans (Billings and Florez 2010; Hunter et al. 2007) and provitamin A levels in maize (Owens et al. 2014). At present, GWAS is one of the most actively researched and applied methods for investigating the genomic underpinnings of Alzheimer’s disease (Wang et al. 2016a), coronary heart disease (Dehghan et al. 2016), Parkinson’s disease (Siitonen et al. 2017), carotenoid biosynthesis in maize (Azmach et al. 2018), and disease resistance in cattle (Coussé et al. 2016), among others. Thus, the ability of GWAS to identify specific genomic regions associated with traits critical for human health and agronomic performance has been demonstrated, and continued refinement of the statistical approaches in GWAS could make this analysis even more relevant for quantitative genetics research and its applications.

The simplest and most widely used analytical approach for GWAS is to perform a separate statistical test for association between each marker and the evaluated trait. For example, a GWAS conducted to identify loci associated with the presence/absence of a disease in humans might perform either a Pearson’s chi-square test or conduct logistic regression separately for every marker in a genome-wide marker set (Nakamura et al. 2012; Wang et al. 2016b). Similarly, a GWAS conducted for a quantitative agronomic trait in a given crop (e.g., Belcher et al. 2018) might use the unified mixed linear model (MLM; Yu et al. 2006) that includes both fixed effect covariates to account for false positives arising from population structure and random effect covariates to account for those arising from familial relatedness.

Although testing each marker individually has been effective in identifying statistically significant marker-trait associations in a wide variety of species and traits, it suffers from two major biological drawbacks. First, the consideration of only one marker at a time makes it impossible to quantify the simultaneous contributions of multiple functional variants located throughout the genome in one statistical model. Second, these single-marker statistical tests typically do not consider the contributions of certain types of non-additive sources of variation, such as epistasis. Improvements to the typical statistical models used for GWAS could lead to more effective models.

Both theoretical (Fisher 1930; Orr 1998) and empirical (Brown et al. 2011; Flint and Mackay 2009; Valdar et al. 2006) quantitative genetics research suggest that quantitative trait variation is under the control of multiple functional variants. Thus, statistical approaches need to complement this by including multiple markers in one model. Stepwise model selection is one of the simplest approaches for simultaneously estimating the additive effects of multiple loci. Here the additive effect of every marker throughout the genome is considered for inclusion as an explanatory variable in an optimal model. An extremely useful application of this approach is the multi-locus mixed-model (MLMM; Segura et al. 2012). In the MLMM, stepwise model selection is conducted on a given set of markers and false positives are controlled for by including the same fixed and random effects covariates as those used in the unified MLM (Yu et al. 2006). An important advantage of the MLMM and similar approaches over single-marker analyses is their capability to substantially lower false positive detection rates of marker/trait associations (Segura et al. 2012). The MLMM has been shown to be useful for GWAS in crop diversity panels, especially as an extra step to further elucidate the signals already identified by an initial genome-wide scan using the unified MLM (Jaiswal et al. 2016; Owens et al. 2014; Rincker et al. 2016).

Another application of stepwise model selection in GWAS is found in the US maize nested association mapping (NAM) panel (Buckler et al. 2009; McMullen et al. 2009; Yu et al. 2008), where it is called joint linkage (JL) analysis. The maize NAM panel consists of 25 recombinant inbred line (RIL) families that share a common parent. To account for the family structure of the NAM panel, JL analysis starts with a baseline model containing the trait of interest as the response variable and the families as a fixed effect. Stepwise model selection is then conducted, where the nested additive effect of each marker within each family is considered for inclusion into an optimal JL analysis model. The use of JL analysis on the US maize NAM population data has proven fruitful for dissecting the genomic sources of many quantitative traits, including flowering time (Buckler et al. 2009), inflorescence (Brown et al. 2011) and leaf blight (Poland et al. 2011). Although the number of markers considered in these studies is orders of magnitude smaller than those found in high-throughput genotypic and/or phenotypic data and the incorporation genomic relatedness on a finer scale could refine the quantification of the identified associations, it is encouraging that JL analysis provided insight into the genetic architecture of those traits. To facilitate its broader adoption in GWAS, JL analysis has been made available in the graphical user interface (GUI) of TASSEL5 (Bradbury et al. 2007), a publicly available Java package.

Non-additive sources of genetic variation are hypothesized to contribute to the discrepancies reported between the observed signals identified in GWAS and what is theoretically expected given the heritability of the trait under study (Zuk et al. 2012). Epistasis, generally defined as the interaction effect between alleles at two or more genomic loci (Phillips 1998), is one such non-additive source. The direct quantification of epistatic effects by inclusion into multi-locus statistical models could improve our understanding of the genomic architecture of traits. A number of statistical approaches have been described for this purpose (e.g., Cordell 2002; Haley and Knott 1992; Jannink and Jansen 2001; Karkkainen et al. 2015) and computationally efficient software has been developed. In particular, FastEpistasis (Schupbach et al. 2010), Glide (Kam-Thong et al. 2012), EpiGPU (Hemani et al. 2011), Boost (Wan et al. 2010), multiEpistSearch (González-Domínguez et al. 2015), and EPIQ (Arkin et al. 2014) explicitly search for pairwise epistasis among a set of markers provided by the user. However, none of the statistical models used in these packages can incorporate contributions from multiple pairs of interacting loci. This is a significant drawback, as a substantial proportion of non-additive variation could be attributable to multiple sets of epistatically interacting loci. In this manuscript we evaluate the Stepwise Procedure for constructing an Additive and Epistatic Multi-Locus model (SPAEML), which could potentially remedy that drawback.

We extended the TASSEL5 code for JL analysis to implement SPAEML and tested its ability to detect additive and epistatic quantitative trait nucleotides (QTNs) as a function of sample size and number of markers. To achieve this, we used genomic data from 2648 individuals from the North Central Regional Plant Introduction Station (NCRPIS) maize diversity panel (Romay et al. 2013) and from an Alzheimer’s disease (AD) case–control cohort consisting of 2099 human subjects (Zou et al. 2012) to simulate traits with different heritabilities and QTN effect sizes. Since these were not nested association mapping populations, the effect of nesting was not enabled in any of our analyses. We compared SPAEML to two other methods. The first, JL analysis, constructs a multi-locus model for additive marker effects and therefore will always misspecify any epistatic markers included in the model as additive. In contrast, FastEpistasis focuses on the interaction effect of one marker pair at a time; thus any additive signals identified by this approach will be misspecified as epistatic. Our hypothesis was that SPAEML can detect and correctly specify both additive and epistatic QTNs.

## Materials and methods

### Stepwise procedure for constructing an additive and epistatic multi-locus model

The statistical approach implemented for SPAEML is similar to those previously described (e.g., Bogdan et al. 2004; Yu et al. 2008). Briefly, this procedure involves identifying the optimal version of the multi-locus linear model that combines additive and epistatic effects:

1 $$Y_i = \mu + \mathop {\sum }\limits_{j \in I} \beta _jx_{ij} + \mathop {\sum }\limits_{\left( {u,v} \right) \in U} \gamma _{uv}x_{iu}x_{iv} + \varepsilon _i$$

for a data set consisting of *n* individuals and *m* markers denoted by *x*_1_,…,*x*_*m*_. In this model, *Y*_*i*_ is the observed trait value of the *i*^th^ individual (e.g., human subject or plant accession); *μ* is the grand mean; *β*_*j*_ is the additive effect of the *j*^th^ marker; *x*_*ij*_ is the observed genotype of the *j*^th^ marker of the *i*^th^ individual, numerically coded as, e.g., 0 for *aa*, 1 for *Aa/aA*, and 2 for *AA*; *γ*_*uv*_ is the two-way epistatic term between the *u*^th^ and the *v*^th^ marker;*x*_*iu*_ and *x*_*iv*_ are the observed genotypes for the *u*^th^ and the *v*^th^ markers, both of which are numerically coded in the same manner as *x*_*ij*_; *I* is a subset of the *m* markers with additive effects included in the model; *U* is another subset of markers with two-way epistatic effects included in the model; and *ε*_*i*_ represents a normally distributed random error term. A stepwise model selection procedure is used to determine the optimal sets of markers belonging to *I* and *U*.

### Simulation study

#### Genotypic and phenotypic data

To evaluate the statistical performance of SPAEML we conducted two independent simulation studies: one using genotypic data from a maize diversity panel, and one using genotypic data from a human case–control study. The maize data were from the NCRPIS maize diversity panel (Romay et al. 2013), consisting of a collection of 2815 diverse maize inbred lines from throughout the world. We focused on a subset of 2648 individuals genotyped for 681,257 single nucleotide polymorphisms (SNPs) using genotyping-by-sequencing (GBS, Elshire et al. 2011). These data are publicly available at: http://cbsusrv04.tc.cornell.edu/users/panzea/filegateway.aspx?category = Genotypes. The second data set is from the Mayo Clinic late-onset Alzheimer’s disease GWAS, which consists of 844 Alzheimer’s disease (AD) cases and 1255 controls (Zou et al. 2012). All 2099 of these individuals were genotyped using 213,528 SNPs located within ±100 kb of 24,526 genes whose transcript levels were measured in Zou et al. (2012). These data are available at: https://www.synapse.org/#!Synapse:syn2910256.

Within each species, we constructed multiple test data sets varying in sample size and number of markers. All test data sets consisted of either the full set of individuals (*n* *=* Max; i.e., 2648 maize or 2099 human individuals), or the same random subset of *n* = 300 individuals in each species. Similarly, the test data sets included either a random subset of *m* = 15,000 SNPs or a random subset of *m* = 5000 SNPs. For both species, all SNPs in the 5000-marker set were also included in the 15,000-marker set.

Traits were simulated as previously described in scheme 2 of Zhang et al. (2010) for each of the above data subsets. First, additive and/or epistatic quantitative trait nucleotides (QTN) were randomly selected from a subset of markers that were present in both the 5000- and 15,000-marker subsets from each species. For consistency across all simulation settings, the range of possible QTN effect sizes was bounded by 0 and 1. A total of five simulation settings were used (Table 1), each with differing numbers of additive and epistatic QTN, their effect sizes, and the broad-sense heritability values (*H*^2^). To empirically evaluate the false positive detection rate of SPAEML in the absence of genomic signals, the traits simulated in the first setting had zero QTN and *H*^2^ = 0. The genomic sources of variation underlying the traits in the next setting consisted of four markers that were randomly selected to be additive QTN and four additional marker pairs that were randomly selected to be epistatic QTN. The additive and epistatic QTN both followed a geometric series; that is, the QTN with the *j*^th^ largest effect size was 0.95^*j*^. Since the purpose of this setting was to evaluate the ability of SPAEML to identify signals for a trait with an ideal genetic architecture, the heritability was set at *H*^2^ = 0.99. To assess whether SPAEML can distinguish between additive and epistatic signals, the next setting consisted of two simulated QTN containing both non-zero additive and non-zero epistatic effects. Thus, the additive effects of these two QTN were 0.90 and 0.81, and the epistatic effect of these two QTN was 0.9. All traits simulated at this setting had a broad-sense heritability of *H*^2^ = 0.95.

**Table 1:** Description of the number of individuals, markers, and genetic architecture considered in the five tested simulation settings

Simulation setting	No. of individuals	No. of markers	Heritability	No. of additive QTN (range of effect sizes)	No. of epistatic QTN (range of effect sizes)
1 = “Null”	300; max^a^	5000 and 15,000	0	0	0
2 = “Ideal”	300; max	5000 and 15,000	0.99	4 (0.81–0.95)	4 (0.81–0.95)
3 = “Additive vs epistatic”	300; max	5000 and 15,000	0.95	2 (0.81–0.90)	1 (0.90)
4 = “Inflorescence-like”	300; max	5000 and 15,000	0.92	26 (9.63 × 10^−10^–0.45)	1 (0.90)
5 = “AD-like”	300; max	5000 and 15,000	0.34	20 (2.75 × 10^−8^–0.9)	1 (0.70)

*QTN* quantitative trait nucleotide, *AD* Alzheimer’s disease

^a^Max, denotes maximum sample size, that is 2648 maize individuals and 2099 human individuals

The next simulation setting strove to emulate the genetic architecture of a trait one might expect to find in a crop species. Thus, traits simulated in this setting were loosely based on the contrasting genetic architecture of inflorescence traits between maize and teosinte (Brown et al. 2011; Doebley et al. 1995). The genetic underpinnings of these simulated traits consisted of one two-way epistatic QTN of effect size 0.90, 26 additive QTN with the effect size of the *j*^th^ QTN set to 0.45^*j*^, and a broad-sense heritability of *H*^2^ = 0.92. In a similar vein, the next setting was based on the genetic architecture of Alzheimer’s disease in humans (Combarros et al. 2009; Medway and Morgan 2014; Wilson et al. 2011). For this setting, a large-effect additive QTN with effect size of 0.90 and a geometric series of 19 additive QTN with the effect size of the *j*^th^ QTN set to 0.40^*j*^ were simulated. In addition, a two-way epistatic QTN with effect size 0.70 was simulated. To imitate the contributions of the *APOE* gene to Alzheimer’s disease (Combarros et al. 2009), one of the two loci contributing to this epistatic QTN was the same as the large-effect additive QTN with effect size of 0.90. Consistent with the literature (Wilson et al. 2011), all traits simulated in this setting had a broad-sense heritability of *H*^2^ = 0.34.

A total of 100 traits were simulated for each setting, species, and sample size. For a given simulated trait and sample size, the cumulative additive and epistatic QTN effects were calculated across all individuals. The variance of these cumulative effects comprised the genetic variance component of the trait. Finally, for a given *H*^2^, we simulated a normal random variable with mean 0 and variance $\sigma _r^2$, where $\sigma _r^2$ is determined from *H*^2^. That is, if we let $\sigma _g^2$ denote the genetic variance component of the trait, then the variance of this normal random variable is calculated by solving the following equation for $\sigma _r^2$:

2 $$H^2 = \frac{{\sigma _g^2}}{{\sigma _g^2 + \sigma _r^2}}$$

Thus, any simulated trait value from a particular individual equals the sum of the cumulative QTN effects and the observed value of the aforementioned normal random variable. For the first setting, with zero QTN and *H*^2^ = 0, this normal random variable was simulated with a variance of $\sigma _r^2 = 1$.

### Statistical models fitted to each trait at each setting

For each trait that was generated in the simulation study, SPAEML, JL analysis, and FastEpistasis were conducted to identify markers exhibiting peak associations with additive and epistatic QTN. For each of the five simulation settings, sample sizes, species, and number of markers, two separate permutation procedures (described in Churchill and Doerge 1994) were conducted 100 times to empirically determine the inclusion and exclusion *P*-value thresholds that control the Type I error rate at 0.05: once for SPAEML and once for JL analysis. We conducted SPAEML using a Java package derived from the original TASSEL5 suite, but with the added ability to include epistasis (https://bitbucket.org/wdmetcalf/tassel-5-threaded-model-fitter); a flowchart of this implementation of SPAEML is provided in Supplementary Figure 1. Additionally, the built-in stepwise model selection procedure from TASSEL was used to conduct the stepwise model selection procedure that only considered additive marker effects, i.e., the procedure which we refer to as JL analysis. The FastEpistasis package was obtained from http://www.vital-it.ch/software/FastEpistasis, and Bonferroni correction was applied to control for multiple testing. FastEpistasis only tests one pair of markers at a time and constructs a model that includes additive effects for each marker and a two-way interaction term that models their epistatic effect.

### Criteria used to quantify the detection of QTNs

For a trait simulated under a given sample size, marker number, species, and setting, a QTN was said to have been detected by one of the three statistical approaches if either a marker contributing to the QTN itself or at least one marker located within a surrounding ±250 kb window was (i) included as a main (additive) effect in SPAEML or JL analysis, (ii) included as part of a two-way interaction (epistatic) effect by SPAEML, or (iii) included as part of a two-way interaction effect with a *P*-value less than or equal to the Bonferroni-adjusted *α* = 0.05 threshold when analyzed in FastEpistasis. Thus, an approach’s (i.e., SPAEML, JL analysis, or FastEpistasis) detection rate of a QTN was defined to be the proportion of the corresponding 100 simulated traits in which a QTN was detected. A similar metric specific to SPAEML, the specification rate of a QTN, was defined as the proportion of 100 traits where an additive QTN was correctly identified by SPAEML as additive, and both loci contributing to an epistatic QTN were correctly identified by SPAEML as epistatic. A window size of ±250 kb has been previously used in maize diversity panels to designate local regions of genomic proximity in maize (Chen and Lipka 2016; Lipka et al. 2013). To enable a side-by-side comparison of results between the two species, the same ±250 kb window size was used in the human data.

A false positive (FP) detection was said to occur for (i) each main effect detected by SPAEML or JL analysis corresponding to a marker located outside of the ±250 kb windows surrounding all QTN, (ii) each two-way interaction effect detected in SPAEML where both corresponding markers were located outside of the ±250 kb windows surrounding all QTN, or when (iii) a statistically significant association outside of these windows was identified by FastEpistasis. Hence the FP rate of a given approach was defined to be the proportion of 100 traits simulated at a given sample size, marker number, species, and setting with at least one FP.

## Results

**Fig. 1:**
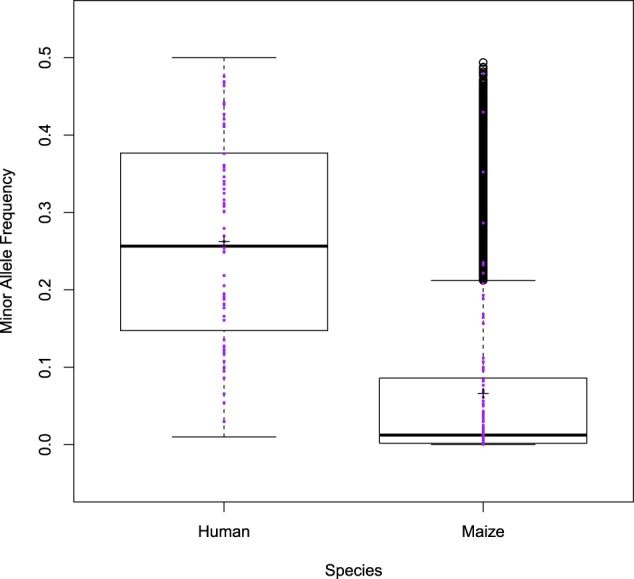
Distribution of the minor allele frequencies (MAFs) of the evaluated single nucleotide polymorphisms (SNPs). Box plots depicting the MAFs (*Y*-axis) of the 15,000 SNPs that were tested in the human data set and the 15,000 SNPs that were tested in the maize data set (*X*-axis). The MAFs of all SNPs that were randomly selected to be quantitative trait nucleotides (QTNs) for the simulation studies are denoted by purple dots. These box plots illustrate that the MAFs of the SNPs in the maize data set tend to be lower than those in the human data set

We conducted a simulation study to explore the impact of sample size and number of markers on the ability of SPAEML to identify additive and epistatic QTN. To enable a thorough investigation, traits with different genetic architectures ranging in complexity were simulated using genotypic data from a maize diversity panel and then again with genotypic data from a human case–control cohort (Table 1). Figure 1 shows that the distributions of minor allele frequencies (MAFs) of the 15,000 markers considered in both species are vastly different. While the majority of the 15,000 SNPs in the maize diversity panel have MAFs below 0.1, the majority of the 15,000 SNPs in the human case–control study have MAFs that are greater than 0.1. Within both data sets, the MAFs of the markers randomly selected to be QTNs span the entire range of observed MAFs. These patterns enabled us to observe the way the collective distribution of allele frequencies in a marker set influenced the performance of SPAEML.

### Observed false positive rates across the five genetic architectures

**Fig. 2:**
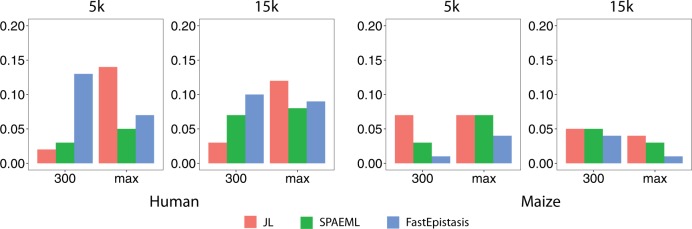
Comparison of false positive rates for the three approaches evaluated in “Null” setting where no quantitative trait nucleotides (QTNs) were simulated. The rate of false positive detection, defined as a SNP located outside of ±250 kb of any of the QTNs, for joint linkage (JL) analysis, the stepwise procedure for constructing an additive and epistatic multi-locus model (SPAEML), and FastEpistasis are plotted on the *Y*-axis of each graph. Starting from the left, the first two graphs show the results for the traits simulated in the human data, while the last two columns show the results for the maize simulated data. The graphs with the title “5k” show the results when 5,000 markers were tested, and the graphs with the title “15k” show the results when 15,000 markers were tested. The *X*-axis of each graph show the sample sizes that were tested, with max indicating the maximum sample size of each data set (2648 in the maize data set and 2099 in the human data set)

The purpose of simulating traits under the “Null” setting was to evaluate the effectiveness of the premutation procedure (used for SPAEML and JL analysis) and the Bonferroni procedure (used for FastEpistasis results) to control the type I error rate at *α* = 0.05. The observed empirical FP rates across species, number of markers, and sample sizes suggest that these procedures are controlling for type I errors reasonably well, with SPAEML having empirical FP rates that are most consistently close to *α* = 0.05 (Fig. 2). The FP rates are generally higher for simulation settings other than “Null,” especially for the traits simulated under the “Ideal” genetic architecture (*H*^2^ = 0.99, all QTNs have large effect; Table 1) in maize (Supplementary Figure 1). These results are not surprising because it is theoretically possible for all three approaches to identify markers that are in linkage disequilibrium (LD) with the simulated QTN. FastEpistasis, which tests the epistatic effect of one pair of loci at a time, tended to yield higher FP rates than the other two stepwise approaches, while SPAEML tended to have low FP rates at the maximum sample sizes in both data sets (Supplementary Figure 2).

### Accuracy of SPAEML at a limited sample size of *n* = 300 individuals

**Fig. 3:**
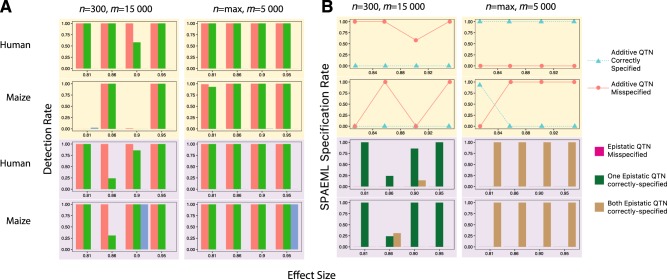
Detection (**a**) and specification (**b**) rates of simulated quantitative trait nucleotides (QTNs) for the three approaches evaluated in the “Ideal” genetic architecture with setting with four large-effect additive QTN and four large-effect epistatic QTN and heritability equal to 0.99 (**a**). The detection rates of the additive QTNs, defined as the proportion of SNPs located within ±250 kb of any of the simulated QTN detected using joint linkage (JL) analysis (red bar), the stepwise procedure for constructing an additive and epistatic multi-locus model (SPAEML; green bar), and FastEpistasis (blue bar) are plotted on the *Y*-axis of each graph. The first two rows (shaded pale yellow) show results for the simulated additive QTN, while the bottom two rows (shaded pale purple) show results for the simulated epistatic QTN. The first and third rows show results for the simulations conducted in the human data set, while the second and fourth rows show results for the simulations conducted in the maize data set. The *X-*axis on each graph depict the effect sizes of the QTN. The left column shows results for *n* = 300 individuals and *m* = 15,000 markers, while the right column shows results for *n* = max individuals (i.e., *n* = 2099 in humans and *n* = 2648 in maize) and *m* = 5000 markers. Both JL and SPAEML are able to detect the additive and epistatic effects, while FastEpistasis failed to detect all the additive effects and most of the epistatic effects. **b** Specification rates of SPAEML, defined as the proportion of times that a detected additive QTN was correctly specified in the SPAEML model as additive, misspecified as epistatic (first two rows); or the proportion of times for a detected epistatic QTN that it was misspecified as additive, only one locus contributing to the QTN was detected, or both loci contributing to the QTN (bottom two rows). These proportions are depicted on the *Y*-axis of each graph. The *X*-axes of each graph, and how they are subdivided into rows and columns, are the same as in **a**. Optimal specification is obtained at *n* = max; *m* = 5000-marker setting and in the human data

The results from these simulation studies show that sample size has a substantially greater impact on QTN detection than the number of markers, underscoring the well-established importance of having sufficient sample sizes when conducting quantitative genetics analysis (Doerge 2002). Nevertheless, to ascertain the limits of the ability of SPAEML to identify genomic signals, all five simulation settings were run with *n* = 300 individuals. One of the most detrimental impacts of small sample size on the accuracy of SPAEML appeared to be on the FP rate; substantially high FP rates from SPAEML were observed only at *n* = 300 (Supplementary Figure 2). In contrast, the FP rates for JL analysis and FastEpistasis were more consistent across sample sizes. At *n* = 300, SPAEML detected QTN at rates vastly superior to those of FastEpistasis, but not as high as those of JL analysis (Fig. 3a; Supplementary Figures 3-10). Finally, we observed that at *n* = 300, SPAEML is more likely to misspecify additive QTN as epistatic and identify only one locus contributing to an epistatic QTN (Fig. 3b; Supplementary Figures 11-18). In contrast at *n* = max, SPAEML yielded (i) minimal FP rates, (ii) QTN detection rates that were comparable to JL analysis, (iii) greater capability to identify both loci underlying epistatic QTN, and (iv) the capacity to distinguish between additive and epistatic signals in traits simulated in the human data set. In light of this contrast and the negligible impact of the number of tested markers on the simulation results, the remaining sections present findings based on *n* = max individuals and *m* = 15,000 markers.

### Distinguishing between additive and epistatic signals at the same locus

Among the three approaches that were evaluated, only the output of SPAEML provide results for both additive and epistatic terms fitted to one model. To characterize the ability of SPAEML to distinguish between additive and epistatic signals, both of the QTNs considered in the “Additive vs. Epistatic” setting harbored non-zero additive and epistatic effects. At this setting, we observed contrasting results between the two species. In maize, SPAEML classified the signals at these QTNs as epistatic 100% of the time, suggesting that SPAEML was unable to distinguish between these additive and epistatic effects (Supplementary Figures 13, 14). Contrastingly, SPAEML identified the additive and epistatic signals underlying both QTN simulated in the human data set for all simulated traits. Similar results were obtained for the Alzheimer’s disease-like (“AD-like”) setting, where the large-effect additive QTN also include a substantially large epistatic signal (Supplementary Figures 17, 18).

### Accuracy in more complex genetic architectures

We compared the accuracy of the three approaches in simulation settings 4 and 5, which approximate the polygenic underpinnings of maize inflorescence (“Inflorescence-like” in Table 1) and Alzheimer’s disease (“AD-like”). Two important characteristics distinguish these two settings. First, “Inflorescence-like” was highly heritable (*H*^2^ = 0.92) while “AD-like” was not (*H*^2^ = 0.34). Secondly, the effect size of the epistatic QTN was substantially higher relative to those of the additive QTN in the “Inflorescence-like” setting, whereas the strength of the epistatic QTN in the “AD-like” setting was not.

**Fig. 4:**
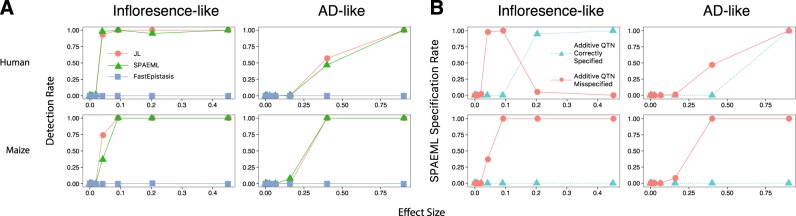
Detection (**a**) and specification (**b**) rates of simulated additive quantitative trait nucleotides (QTNs) for the three approaches evaluated in the two complex genetic architectures at a maximum number of individuals (*n* = 2099 human subjects and *n* = 2648 maize lines) and 15,000 markers (**a**) The detection rates of the additive QTNs, defined as the proportion of SNPs located within ±250 kb of any of the simulated QTNs detected using joint linkage (JL) analysis, the stepwise procedure for constructing an additive and epistatic multi-locus model (SPAEML), and FastEpistasis are plotted on the *Y*-axis of each graph. The first row shows results for the simulations conducted in the human data set, while the second row shows results for the simulations conducted in the maize data set. The *X*-axis on each graph depict the effect sizes of the additive QTN. The left column shows results for the inflorescence-like genetic architecture, while the right column shows results for the AD-like genetic architecture. Similar detection rates were observed across JL analysis and SPAEML, while FastEpistasis failed to detect all the additive effects. **b** Specification rates of SPAEML, defined as the proportion of times that a detected additive QTN was correctly specified in the SPAEML model as additive or misspecified as epistatic, are depicted on the *Y*-axis of each graph. The *X*-axes of each graph, and how they are subdivided into rows and columns, are the same as in **a**. Correct specification of additive QTN occurs in the traits simulated using human data. “Inflorescence-like” = setting with 26 additive QTN, one epistatic QTN and heritability equal to 0.92; “AD-like” = setting with 20 additive QTN, one epistatic QTN and heritability = 0.34

The detection rate for additive QTN improved as a function of the effect size for both JL and SPAEML, with roughly comparable accuracy between the two approaches (Fig. 4a). In the human data set, SPAEML provided the added advantage of always correctly identifying both loci contributing to the epistatic QTN, and correctly specifying additive QTNs as a function of their effect size (Fig. 4a and Supplementary Figures 17, 18). This latter result intuitively makes sense: the stronger the QTN effect, the more likely it is to be distinguished from a non-additive signal. Among the corresponding traits simulated with maize data, SPAEML was at most capable of detecting one out of two loci contributing to an epistatic QTN, and all additive QTNs were misspecified as epistatic (Fig. 4b). We hypothesize that the generally lower MAF observed in the markers from the maize data set provided weaker statistical support for each of the simulated QTN, resulting in the observed misspecification.

## Discussion

Statistical approaches that consider the additive and epistatic contributions of multiple genomic loci could enable unprecedented quantification of the genetic architecture of agronomically important and human health-related quantitative traits. Using genotypic data from a maize diversity panel and a case–control study of Alzheimer’s disease in humans, we conducted a simulation study to determine the accuracy and limits of applicability of SPAEML. Specifically, we assessed the impact of sample size, number of markers, MAF, and the genetic architecture underlying a given trait on the ability of SPAEML to detect and correctly specify additive and epistatic QTN. Our results suggest that sample size has greater influence on the performance of SPAEML than the number of markers, in all considered cases. Additionally, the capability of SPAEML to distinguish between additive and epistatic QTN was much greater when traits were simulated in the human data set, possibly due to the generally higher values of marker MAFs. At the maximum evaluated sample sizes, the detection rate of SPAEML was comparable to JL analysis, and unequivocally superior to that of FastEpistasis.

Our study builds upon previous work (Haley and Knott 1992; Jannink and Jansen 2001; Karkkainen et al. 2015; Sehgal et al 2017) that explicitly assesses the ability of stepwise-based or similar approaches to identify and distinguish between additive and epistatic genomic signals. Novel state-of-the-art computational approaches (Gittens et al. 2016) and inexpensive genotyping protocols (Elshire et al. 2011; Hohenlohe et al. 2010) are resulting in extremely large amounts of genotypic and phenotypic data. Larger sample sizes facilitate improved accuracy of analyses. However, exhaustive searches for multiple sets of epistatically interacting loci on a genome-wide scale in large data sets faces a difficult multiple-testing problem (Karkkainen et al. 2015). Stepwise model selection and related approaches have been successful in circumventing this problem in the past (Brown et al. 2011; Mathew et al. 2018; Tian et al. 2011) by considering a relatively small number of total markers in their analyses; this past success drove us to investigate SPAEML.

Based on our results, we expect SPAEML will be particularly useful for quantifying additive and epistatic marker-trait associations in specific genomic regions that have been identified in a priori biological or statistical analyses. This will result in the analysis of a smaller set of markers, thus yielding a smaller search space for optimal models and enabling researchers to capitalize on the accuracy of SPAEML that we demonstrate here. Our exploration of the factors that influence the accuracy of SPAEML is not exhaustive, but is sufficient to complement so-called “search space reduction” efforts (Ritchie 2011; Wei et al. 2014) by providing a rough assessment of the number of markers to target within the genomic regions identified in a priori analyses.

### Effect of sample size

Our study confirmed the common expectation that sample size positively affects the accuracy of SPAEML. We also demonstrated that SPAEML is capable of true positive detection and even correct specification of additive and epistatic QTN at the smaller sample size that we explored (*n* = 300). However, the accuracy improves dramatically at larger sample sizes. Although this result is unsurprising, direct quantification of SPAEML’s ability to identify additive and epistatic QTN at different sample sizes is informative, as it is useful to know how a model will behave on a smaller data set when desired sample sizes are unavailable. Our results show that even in those cases SPAEML will find many significant SNPs and epistatic pairs, although they may be misspecified in the final model.

### Effect of the marker set size

In contrast to the substantial impact of sample size on the accuracy of SPAEML, we observed similar true and false positive rates at the two marker sizes that were tested. From a statistical perspective, these results suggest that for these simulated data, the conservativeness of the multiple testing problem is similar for both 5000 and 15,000 markers. Thus, the larger marker set does not decrease the accuracy of SPAEML. This is important, as our marker sets are orders of magnitude smaller than those currently available on a genome-wide scale in heavily researched species. We hope the usefulness of SPAEML holds for larger sets, although direct extrapolation is not recommended. We believe this method is best used on a set of markers that has been whittled down by using prior biological information, such as linkage disequilibrium analysis, hypothetical relations between markers and cellular pathways, or other preliminary analyses that remove markers that do not contribute to the final model. This will bring the problem into the setting of optimal performance for SPAEML, and also reduce the computational burden from testing both additive and epistatic effects, which grows binomially with the marker set size.

### Effect of the minor allele frequency

We found SPAEML to be much more capable of distinguishing between additive and epistatic signals for traits simulated in the human data set, despite that the same number of markers, similar number of individuals, and the same simulated genetic architectures were evaluated in the maize and human data sets. We propose two distinct but not mutually exclusive hypotheses to explain these results. First, differences between the underlying characteristics of the maize and human genomes could result in LD-related properties being more favorable for SPAEML to work optimally in the human data set. The second hypothesis is that the differences in accuracy are a downstream ramification of the difference in MAF distribution across the two data sets (Fig. 1), potentially explained by the procedures for data collection. The maize data set is a diversity panel, meaning that it consists of a wide variety of genetically diverse species (Romay et al. 2013). Thus rare variants are prominent, and consequently SNPs with low MAFs are observed. Although rare variants are undoubtedly also present in the human genome, recent research suggests that the humans tend to be far less genetically diverse than plants, having gone through multiple rounds of purifying selection during inter-continental migrations in human evolution (Reich 2018; Schlebusch and Jakobsson 2018). Combined with the fact that the human data we analyzed were from a case–control study, low MAFs are less prominent. In any case, the differences in SPAEML accuracy suggest that both the genomic characteristics of a species and the distribution of MAFs among the tested markers could exhibit a critical impact on the results.

### Conclusions and next steps

To ensure that the most appropriate biological conclusions are made by breeding, medical, and quantitative genetics research communities, it is imperative that statistical models which approximate the genetic architecture of traits are accurate. By design, both JL analysis and FastEpistasis oversimplify the intricate patterns of main effects and multifaceted interactions between loci contributing to phenotypic variability. While JL is designed to only consider additive effects in a multi-locus model, FastEpistasis is designed to only test for epistasis, one pair of markers at a time. In contrast, we demonstrate that SPAEML is a sensitive and accurate approach capable of identifying and distinguishing between additive and epistatic genomic signals, at least for data sets of several thousand samples and markers. We suggest that SPAEML, which conducts model selection for all possible main effects and two-way interaction effects of a set of markers, is best used for constructing an accurate model on a limited set of markers identified through an a priori analysis, once markers in genomic regions with no contributions to phenotypic variation have already been eliminated. We hypothesize that utilizing as much a priori information as possible on both the target trait and data set, particularly transcriptomic, metabolomic and genomic relatedness between individuals, could theoretically expedite the identification of such genomic regions.

### Data archiving

The Java code used to perform SPAEML is available for download at https://bitbucket.org/wdmetcalf/tassel-5-threaded-model-fitter. The genotypic data, simulated trait data, and code to simulate the traits are available at: https://github.com/ncsa/EpiQuant_GWAS_Simulations.

## Electronic supplementary material

Supplementary Figure Captions

Supplementary Figure 1

Supplementary Figure 2

Supplementary Figure 3

Supplementary Figure 4

Supplementary Figure 5

Supplementary Figure 6

Supplementary Figure 7

Supplementary Figure 8

Supplementary Figure 9

Supplementary Figure 10

Supplementary Figure 11

Supplementary Figure 12

Supplementary Figure 13

Supplementary Figure 14

Supplementary Figure 15

Supplementary Figure 16

Supplementary Figure 17

Supplementary Figure 18
