# Supplementary material for: An assessment of true and false positive detection rates of stepwise epistatic model selection as a function of sample size and number of markers
Source: Heredity (Edinb). 2018 Nov 15;122(5):660–71. doi: 10.1038/s41437-018-0162-2 (PMC6462028; doi:10.1038/s41437-018-0162-2)
Supplement: Supplementary file 1 — Supplementary Figure Captions [file 41437_2018_162_MOESM1_ESM.doc]

Supplementary Figure 1. Flowchart summarizing the implementation of the stepwise procedure for constructing an additive and epistatic multi-locus model (SPAEML) in a java-based software package derived from TASSEL5. The boxes with a blue outline highlight the forward model selection steps, while the boxes with an orange outline highlight the backward model selection steps.

Supplementary Figure 2. Comparison of false positive rates for the three approaches evaluated in the simulation studies. The rate of false positive detection, defined as a SNP located outside of +/- 250 kb of any of the simulated quantitative trait nucleotides (QTNs), for joint linkage (JL) analysis, the stepwise procedure for constructing an additive and epistatic multi-locus model (SPAEML), and FastEpistasis are plotted on the Y-axis of each graph. The rows depict the genetic architecture that was simulated, while the columns depict the number of markers that were evaluated. The first two columns show the results for the traits simulated in the human data, while the last two columns show the results for the maize simulated data. The X-axis of each graph show the sample sizes that were tested, with max indicating the maximum sample size of each dataset (that is, *n* = 2,648 in the maize dataset and *n*  = 2,099 in the human dataset). In general, FastEpistasis tended to yield higher false positive rates than the other two approaches, and SPAEML tended to have low false positive rates at the maximum sample sizes in both datasets. Null = setting with no simulated QTNs, Ideal = setting with four large-effect additive QTN and four large-effect epistatic QTN and heritability equal to 0.99, Add.vs.Epi. = setting with two QTN, both of which have non-zero additive and epistatic effects and heritability equal to 0.95, Inflorescence-like = setting with 26 additive QTN, one epistatic QTN and heritability equal to 0.92, AD-like = setting with 20 additive QTN, one epistatic QTN and heritability = 0.34, 5k = analyses conducted using 5,000 markers, 15k = analyses conducted using 15,000 markers.

Supplementary Figure 3. Detection rates of simulated quantitative trait nucleotides (QTNs) for the three approaches evaluated in two of the simulated genetic architectures simulated with all *n* = 300 maize individuals, *n* = 300 human individuals and 5,000 markers in both datasets. The detection rates, defined as a SNP located within +/- 250 kb of any of the simulated QTN, for joint linkage (JL) analysis, the stepwise procedure for constructing an additive and epistatic multi-locus model (SPAEML), and FastEpistasis are plotted on the Y-axis of each graph. The first two rows show results for the simulated additive QTN, while the bottom two rows show results for the simulated epistatic QTN. The first and third rows show results for the simulations conducted in the human dataset, while the second and fourth rows show results for the simulations conducted in the maize dataset. The X-axis on each graph depict the effect sizes of the QTN. Both JL and SPAEML are able to detect the additive and epistatic effects, while FastEpistasis failed to detect all the additive effects and most of the epistatic effects. Ideal = setting with four large-effect additive QTN and four large-effect epistatic QTN and heritability equal to 0.99, Add.vs.Epi. = setting with two QTN, both of which have non-zero additive and epistatic effects and heritability equal to 0.95.

Supplementary Figure 4. Detection rates of simulated quantitative trait nucleotides (QTNs) for the three approaches evaluated in two of the simulated genetic architectures simulated with all *n* = 300 maize individuals, *n* = 300 human individuals and 15,000 markers in both datasets. The detection rates, defined as a SNP located within +/- 250 kb of any of the simulated QTN, for joint linkage (JL) analysis, the stepwise procedure for constructing an additive and epistatic multi-locus model (SPAEML), and FastEpistasis are plotted on the Y-axis of each graph. The first two rows show results for the simulated additive QTNs, while the bottom two rows show results for the simulated epistatic QTN. The first and third rows show results for the simulations conducted in the human dataset, while the second and fourth rows show results for the simulations conducted in the maize dataset. The X-axis on each graph depict the effect sizes of the QTN. Both JL and SPAEML are able to detect the additive and epistatic effects, while FastEpistasis failed to detect the majority of the additive effects and most of the epistatic effects. Ideal = setting with four large-effect additive QTN and four large-effect epistatic QTN and heritability equal to 0.99, Add.vs.Epi. = setting with two QTN, both of which have non-zero additive and epistatic effects and heritability equal to 0.95.

Supplementary Figure 5. Detection rates of simulated quantitative trait nucleotides (QTNs) for the three approaches evaluated in two of the simulated genetic architectures simulated with all *n* = 2,648 maize individuals, *n* = 2,099 human individuals and 5,000 markers in both datasets. The detection rates, defined as a SNP located within +/- 250 kb of any of the simulated QTN, for joint linkage (JL) analysis, the stepwise procedure for constructing an additive and epistatic multi-locus model (SPAEML), and FastEpistasis are plotted on the Y-axis of each graph. The first two rows show results for the simulated additive QTN, while the bottom two rows show results for the simulated epistatic QTN. The first and third rows show results for the simulations conducted in the human dataset, while the second and fourth rows show results for the simulations conducted in the maize dataset. The X-axis on each graph depict the effect sizes of the QTN. Both JL and SPAEML are able to detect the additive and epistatic effects, while FastEpistasis failed to detect the vast majority of the additive effects and most of the epistatic effects. Ideal = setting with four large-effect additive QTN and four large-effect epistatic QTN and heritability equal to 0.99, Add.vs.Epi. = setting with two QTN, both of which have non-zero additive and epistatic effects and heritability equal to 0.95.

Supplementary Figure 6. Detection rates of simulated quantitative trait nucleotides (QTNs) for the three approaches evaluated in two of the simulated genetic architectures simulated with all *n* = 2,648 maize individuals, *n* = 2,099 human individuals and 15,000 markers in both datasets. The detection rates, defined as a SNP located within +/- 250 kb of any of the simulated QTN, for joint linkage (JL) analysis, the stepwise procedure for constructing an additive and epistatic multi-locus model (SPAEML), and FastEpistasis are plotted on the Y-axis of each graph. The first two rows show results for the simulated additive QTN, while the bottom two rows show results for the simulated epistatic QTN. The first and third rows show results for the simulations conducted in the human dataset, while the second and fourth rows show results for the simulations conducted in the maize dataset. The X-axis on each graph depict the effect sizes of the QTN. Both JL and SPAEML are able to detect the additive and epistatic effects, while FastEpistasis failed to detect all the additive effects and most of the epistatic effects. Ideal = setting with four large-effect additive QTN and four large-effect epistatic QTN and heritability equal to 0.99, Add.vs.Epi. = setting with two QTN, both of which have non-zero additive and epistatic effects and heritability equal to 0.95.

Supplementary Figure 7. Detection rates of simulated quantitative trait nucleotides (QTNs) for the three approaches evaluated in two of the simulated genetic architectures simulated with all *n* = 300 maize individuals, *n* = 300 human individuals and 5,000 markers in both datasets. The detection rates, defined as a SNP located within +/- 250 kb of any of the simulated QTN, for joint linkage (JL) analysis, the stepwise procedure for constructing an additive and epistatic multi-locus model (SPAEML), and FastEpistasis are plotted on the Y-axis of each graph. The first two rows show results for the simulated additive QTN, while the bottom two rows show results for the simulated epistatic QTN. The first and third rows show results for the simulations conducted in the human dataset, while the second and fourth rows show results for the simulations conducted in the maize dataset. The X-axis on each graph depict the effect sizes of the QTN. Both JL and SPAEML are able to detect the additive and epistatic effects, while FastEpistasis failed to detect the vast majority of the additive effects and most of the epistatic effects. Inflorescence-like = setting with 26 additive QTN, one epistatic QTN and heritability equal to 0.92, AD-like = setting with 20 additive QTN, one epistatic QTN and heritability = 0.34.

Supplementary Figure 8. Detection rates of simulated quantitative trait nucleotides (QTNs) for the three approaches evaluated in two of the simulated genetic architectures simulated with all *n* = 300 maize individuals, *n* = 300 human individuals and 15,000 markers in both datasets. The detection rates, defined as a SNP located within +/- 250 kb of any of the simulated QTN, for joint linkage (JL) analysis, the stepwise procedure for constructing an additive and epistatic multi-locus model (SPAEML), and FastEpistasis are plotted on the Y-axis of each graph. The first two rows show results for the simulated additive QTN, while the bottom two rows show results for the simulated epistatic QTN. The first and third rows show results for the simulations conducted in the human dataset, while the second and fourth rows show results for the simulations conducted in the maize dataset. The X-axis on each graph depict the effect sizes of the QTN. Both JL and SPAEML are able to detect the additive and epistatic effects, while FastEpistasis failed to detect all the additive effects and most of the epistatic effects. Inflorescence-like = setting with 26 additive QTN, one epistatic QTN and heritability equal to 0.92, AD-like = setting with 20 additive QTN, one epistatic QTN and heritability = 0.34.

Supplementary Figure 9. Detection rates of simulated quantitative trait nucleotides (QTNs) for the three approaches evaluated in two of the simulated genetic architectures simulated with all *n* = 2,648 maize individuals, *n* = 2,099 human individuals and 5,000 markers in both datasets. The detection rates, defined as a SNP located within +/- 250 kb of any of the simulated QTN, for joint linkage (JL) analysis, the stepwise procedure for constructing an additive and epistatic multi-locus model (SPAEML), and FastEpistasis are plotted on the Y-axis of each graph. The first two rows show results for the simulated additive QTN, while the bottom two rows show results for the simulated epistatic QTN. The first and third rows show results for the simulations conducted in the human dataset, while the second and fourth rows show results for the simulations conducted in the maize dataset. The X-axis on each graph depict the effect sizes of the QTN. Both JL and SPAEML are able to detect the additive and epistatic effects, while FastEpistasis failed to detect all the additive effects. Inflorescence-like = setting with 26 additive QTN, one epistatic QTN and heritability equal to 0.92, AD-like = setting with 20 additive QTN, one epistatic QTN and heritability = 0.34.

Supplementary Figure 10. Detection rates of simulated quantitative trait nucleotides (QTNs) for the three approaches evaluated in two of the simulated genetic architectures simulated with all *n* = 2,648 maize individuals, *n* = 2,099 human individuals and 15,000 markers in both datasets. The detection rates, defined as a SNP located within +/- 250 kb of any of the simulated QTN, for joint linkage (JL) analysis, the stepwise procedure for constructing an additive and epistatic multi-locus model (SPAEML), and FastEpistasis are plotted on the Y-axis of each graph. The first two rows show results for the simulated additive QTN, while the bottom two rows show results for the simulated epistatic QTN. The first and third rows show results for the simulations conducted in the human dataset, while the second and fourth rows show results for the simulations conducted in the maize dataset. The X-axis on each graph depict the effect sizes of the QTN. Both JL and SPAEML are able to detect the additive and epistatic effects, while FastEpistasis failed to detect all the additive effects. Inflorescence-like = setting with 26 additive QTN, one epistatic QTN and heritability equal to 0.92, AD-like = setting with 20 additive QTN, one epistatic QTN and heritability = 0.34.

Supplementary Figure 11. Stepwise procedure for constructing an additive and epistatic multi-locus model (SPAEML) specification rates of simulated quantitative trait nucleotides (QTNs) in two of the genetic architectures simulated with all *n* = 300 maize individuals, *n* = 300 human individuals and 5,000 markers in each dataset. The first two rows depict the proportion of times that an additive QTN was correctly specified as an additive main effect by SPAEML (blue line) and misspecified as one of two markers in a two-way epistatic interaction effect (red line). The bottom two rows depict the proportion of times both loci contributing to an epistatic QTN were detected as a two-way interaction effect (blue bar), the proportion of times that only one locus from an epistatic QTN was detected as a two-way interaction effect (green bar), and the proportion of times that an epistatic QTN was misspecified as additive effect (red bar). The first and third rows show the results for traits simulated in the human data, while the second and fourth rows show the results for traits simulated in the maize data. The X-axis on each graph depict the effect sizes of the QTN. These results suggest that SPAEML is more amenable to correctly specifying additive QTN as additive and identifying both loci contributing to epistatic QTN in the human dataset. Ideal = setting with four large-effect additive QTN and four large-effect epistatic QTN and heritability equal to 0.99, Add.vs.Epi. = setting with two QTN, both of which have non-zero additive and epistatic effects and heritability equal to 0.95.

Supplementary Figure 12. Stepwise procedure for constructing an additive and epistatic multi-locus model (SPAEML) specification rates of simulated quantitative trait nucleotides (QTNs) in two of the genetic architectures simulated with all *n* = 300 maize individuals, *n* = 300 human individuals and 15,000 markers in each dataset. The first two rows depict the proportion of times that an additive QTN was correctly specified as an additive main effect by SPAEML (blue line) and misspecified as one of two markers in a two-way epistatic interaction effect (red line). The bottom two rows depict the proportion of times both loci contributing to an epistatic QTN were detected as a two-way interaction effect (blue bar), the proportion of times that only one locus from an epistatic QTN was detected as a two-way interaction effect (green bar), and the proportion of times that an epistatic QTN was misspecified as additive effect (red bar). The first and third rows show the results for traits simulated in the human data, while the second and fourth rows show the results for traits simulated in the maize data. The X-axis on each graph depict the effect sizes of the QTN. These results suggest that SPAEML is more amenable to correctly specifying additive QTN as additive and identifying both loci contributing to epistatic QTN in the human dataset. Ideal = setting with four large-effect additive QTN and four large-effect epistatic QTN and heritability equal to 0.99, Add.vs.Epi. = setting with two QTN, both of which have non-zero additive and epistatic effects and heritability equal to 0.95.

Supplementary Figure 13. Stepwise procedure for constructing an additive and epistatic multi-locus model (SPAEML) specification rates of simulated quantitative trait nucleotides (QTNs) in two of the genetic architectures simulated with all *n* = 2,648 maize individuals, *n* = 2,099 human individuals and 5,000 markers in each dataset. The first two rows depict the proportion of times that an additive QTN was correctly specified as an additive main effect by SPAEML (blue line) and misspecified as one of two markers in a two-way epistatic interaction effect (red line). The bottom two rows depict the proportion of times both loci contributing to an epistatic QTN were detected as a two-way interaction effect (blue bar), the proportion of times that only one locus from an epistatic QTN was detected as a two-way interaction effect (green bar), and the proportion of times that an epistatic QTN was misspecified as additive effect (red bar). The first and third rows show the results for traits simulated in the human data, while the second and fourth rows show the results for traits simulated in the maize data. The X-axis on each graph depict the effect sizes of the QTN. These results suggest that SPAEML is more amenable to correctly specifying additive QTN as additive and identifying both loci contributing to epistatic QTN in the human dataset. Ideal = setting with four large-effect additive QTN and four large-effect epistatic QTN and heritability equal to 0.99, Add.vs.Epi. = setting with two QTN, both of which have non-zero additive and epistatic effects and heritability equal to 0.95.

Supplementary Figure 14. Stepwise procedure for constructing an additive and epistatic multi-locus model (SPAEML) specification rates of simulated quantitative trait nucleotides (QTNs) in two of the genetic architectures simulated with all *n* = 2,648 maize individuals, *n* = 2,099 human individuals and 15,000 markers in each dataset. The first two rows depict the proportion of times that an additive QTN was correctly specified as an additive main effect by SPAEML (blue line) and misspecified as one of two markers in a two-way epistatic interaction effect (red line). The bottom two rows depict the proportion of times both loci contributing to an epistatic QTN were detected as a two-way interaction effect (blue bar), the proportion of times that only one locus from an epistatic QTN was detected as a two-way interaction effect (green bar), and the proportion of times that an epistatic QTN was misspecified as additive effect (red bar). The first and third rows show the results for traits simulated in the human data, while the second and fourth rows show the results for traits simulated in the maize data. The X-axis on each graph depict the effect sizes of the QTN. These results suggest that SPAEML is more amenable to correctly specifying additive QTN as additive and identifying both loci contributing to epistatic QTN in the human dataset. Ideal = setting with four large-effect additive QTN and four large-effect epistatic QTN and heritability equal to 0.99, Add.vs.Epi. = setting with two QTN, both of which have non-zero additive and epistatic effects and heritability equal to 0.95.

Supplementary Figure 15. Stepwise procedure for constructing an additive and epistatic multi-locus model (SPAEML) specification rates of simulated quantitative trait nucleotides (QTNs) in two of the genetic architectures simulated with all *n* = 300 maize individuals, *n* = 300 human individuals and 5,000 markers in each dataset. The first two rows depict the proportion of times that an additive QTN was correctly specified as an additive main effect by SPAEML (blue line) and misspecified as one of two markers in a two-way epistatic interaction effect (red line). The bottom two rows depict the proportion of times both loci contributing to an epistatic QTN were detected as a two-way interaction effect (blue bar), the proportion of times that only one locus from an epistatic QTN was detected as a two-way interaction effect (green bar), and the proportion of times that an epistatic QTN was misspecified as additive effect (red bar). The first and third rows show the results for traits simulated in the human data, while the second and fourth rows show the results for traits simulated in the maize data. The X-axis on each graph depict the effect sizes of the QTN. These results suggest that SPAEML is more amenable to correctly specifying additive QTN as additive and identifying both loci contributing to epistatic QTN in the human dataset. Inflorescence-like = setting with 26 additive QTN, one epistatic QTN and heritability equal to 0.92, AD-like = setting with 20 additive QTN, one epistatic QTN and heritability = 0.34.

Supplementary Figure 16. Stepwise procedure for constructing an additive and epistatic multi-locus model (SPAEML) specification rates of simulated quantitative trait nucleotides (QTNs) in two of the genetic architectures simulated with all *n* = 300 maize individuals, *n* = 300 human individuals and 15,000 markers in each dataset. The first two rows depict the proportion of times that an additive QTN was correctly specified as an additive main effect by SPAEML (blue line) and misspecified as one of two markers in a two-way epistatic interaction effect (red line). The bottom two rows depict the proportion of times both loci contributing to an epistatic QTN were detected as a two-way interaction effect (blue bar), the proportion of times that only one locus from an epistatic QTN was detected as a two-way interaction effect (green bar), and the proportion of times that an epistatic QTN was misspecified as additive effect (red bar). The first and third rows show the results for traits simulated in the human data, while the second and fourth rows show the results for traits simulated in the maize data. The X-axis on each graph depict the effect sizes of the QTN. These results suggest that SPAEML is more amenable to correctly specifying additive QTN as additive and identifying both loci contributing to epistatic QTN in the human dataset. Inflorescence-like = setting with 26 additive QTN, one epistatic QTN and heritability equal to 0.92, AD-like = setting with 20 additive QTN, one epistatic QTN and heritability = 0.34.

Supplementary Figure 17. Stepwise procedure for constructing an additive and epistatic multi-locus model (SPAEML) specification rates of simulated quantitative trait nucleotides (QTNs) in two of the genetic architectures simulated with all *n* = 2,648 maize individuals, *n* = 2,099 human individuals and 5,000 markers in each dataset. The first two rows depict the proportion of times that an additive QTN was correctly specified as an additive main effect by SPAEML (blue line) and misspecified as one of two markers in a two-way epistatic interaction effect (red line). The bottom two rows depict the proportion of times both loci contributing to an epistatic QTN were detected as a two-way interaction effect (blue bar), the proportion of times that only one locus from an epistatic QTN was detected as a two-way interaction effect (green bar), and the proportion of times that an epistatic QTN was misspecified as additive effect (red bar). The first and third rows show the results for traits simulated in the human data, while the second and fourth rows show the results for traits simulated in the maize data. The X-axis on each graph depict the effect sizes of the QTN. These results suggest that SPAEML is more amenable to correctly specifying additive QTN as additive and identifying both loci contributing to epistatic QTN in the human dataset. Inflorescence-like = setting with 26 additive QTN, one epistatic QTN and heritability equal to 0.92, AD-like = setting with 20 additive QTN, one epistatic QTN and heritability = 0.34.

Supplementary Figure 18. Stepwise procedure for constructing an additive and epistatic multi-locus model (SPAEML) specification rates of simulated quantitative trait nucleotides (QTNs) in two of the genetic architectures simulated with all *n* = 2,648 maize individuals, *n* = 2,099 human individuals and 15,000 markers in each dataset. The first two rows depict the proportion of times that an additive QTN was correctly specified as an additive main effect by SPAEML (blue line) and misspecified as one of two markers in a two-way epistatic interaction effect (red line). The bottom two rows depict the proportion of times both loci contributing to an epistatic QTN were detected as a two-way interaction effect (blue bar), the proportion of times that only one locus from an epistatic QTN was detected as a two-way interaction effect (green bar), and the proportion of times that an epistatic QTN was misspecified as additive effect (red bar). The first and third rows show the results for traits simulated in the human data, while the second and fourth rows show the results for traits simulated in the maize data. The X-axis on each graph depict the effect sizes of the QTN. These results suggest that SPAEML is more amenable to correctly specifying additive QTN as additive and identifying both loci contributing to epistatic QTN in the human dataset. Inflorescence-like = setting with 26 additive QTN, one epistatic QTN and heritability equal to 0.92, AD-like = setting with 20 additive QTN, one epistatic QTN and heritability = 0.34.
