# Supplementary figures and images for: An assessment of true and false positive detection rates of stepwise epistatic model selection as a function of sample size and number of markers
Source: Heredity (Edinb). 2018 Nov 15;122(5):660–71. doi: 10.1038/s41437-018-0162-2 (PMC6462028; doi:10.1038/s41437-018-0162-2)

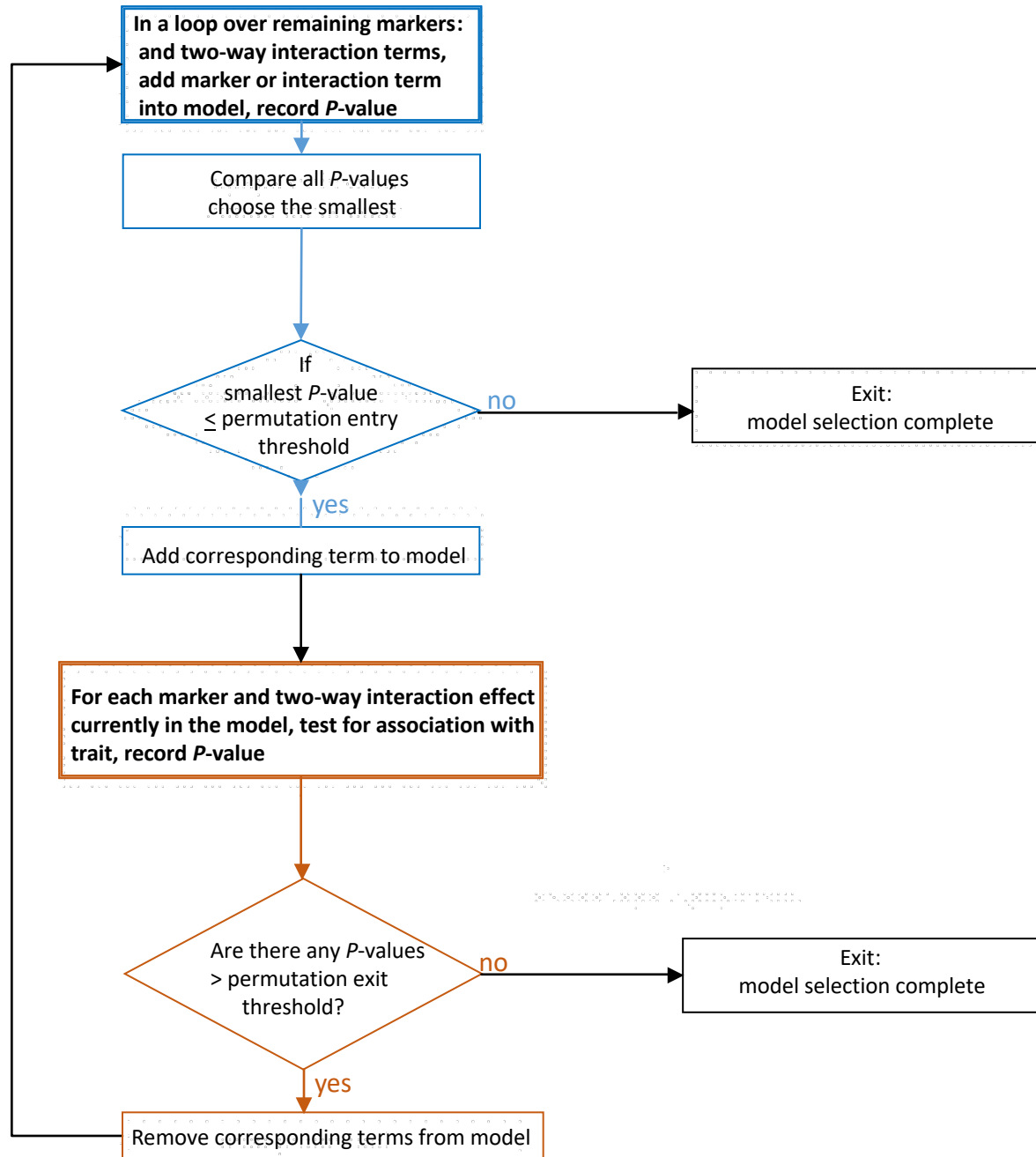

Supplement: Supplementary file 2 — Supplementary Figure 1 [file 41437_2018_162_MOESM2_ESM.pdf]

# Maize

## Ideal

## Add. vs. Epi.

Inflorescence  
-like

AD-like

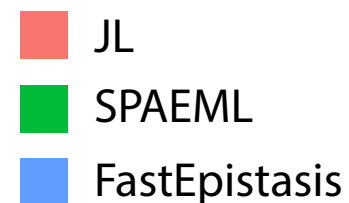

Supplement: Supplementary file 3 — Supplementary Figure 2 [file 41437_2018_162_MOESM3_ESM.pdf]

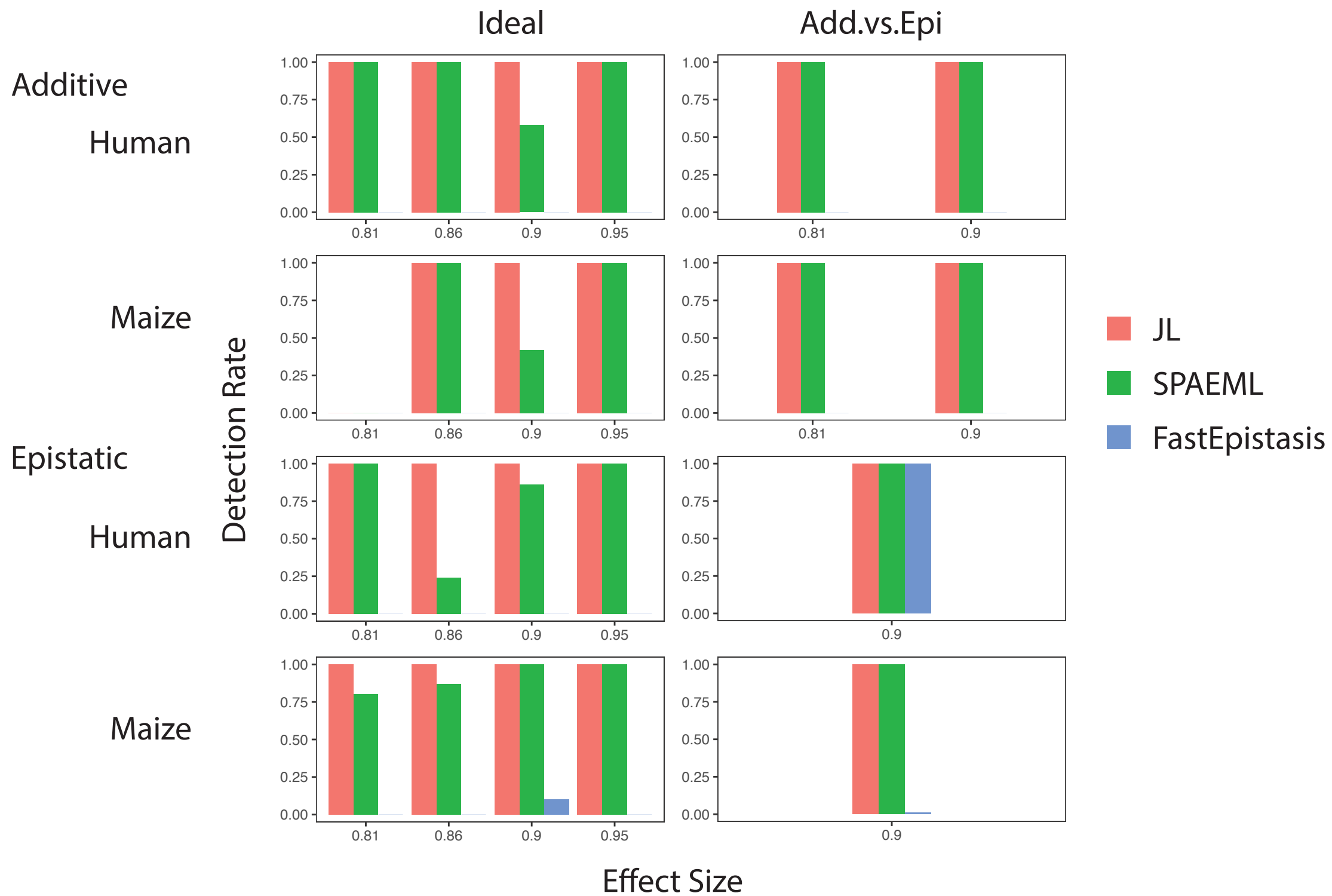

Supplement: Supplementary file 4 — Supplementary Figure 3 [file 41437_2018_162_MOESM4_ESM.pdf]

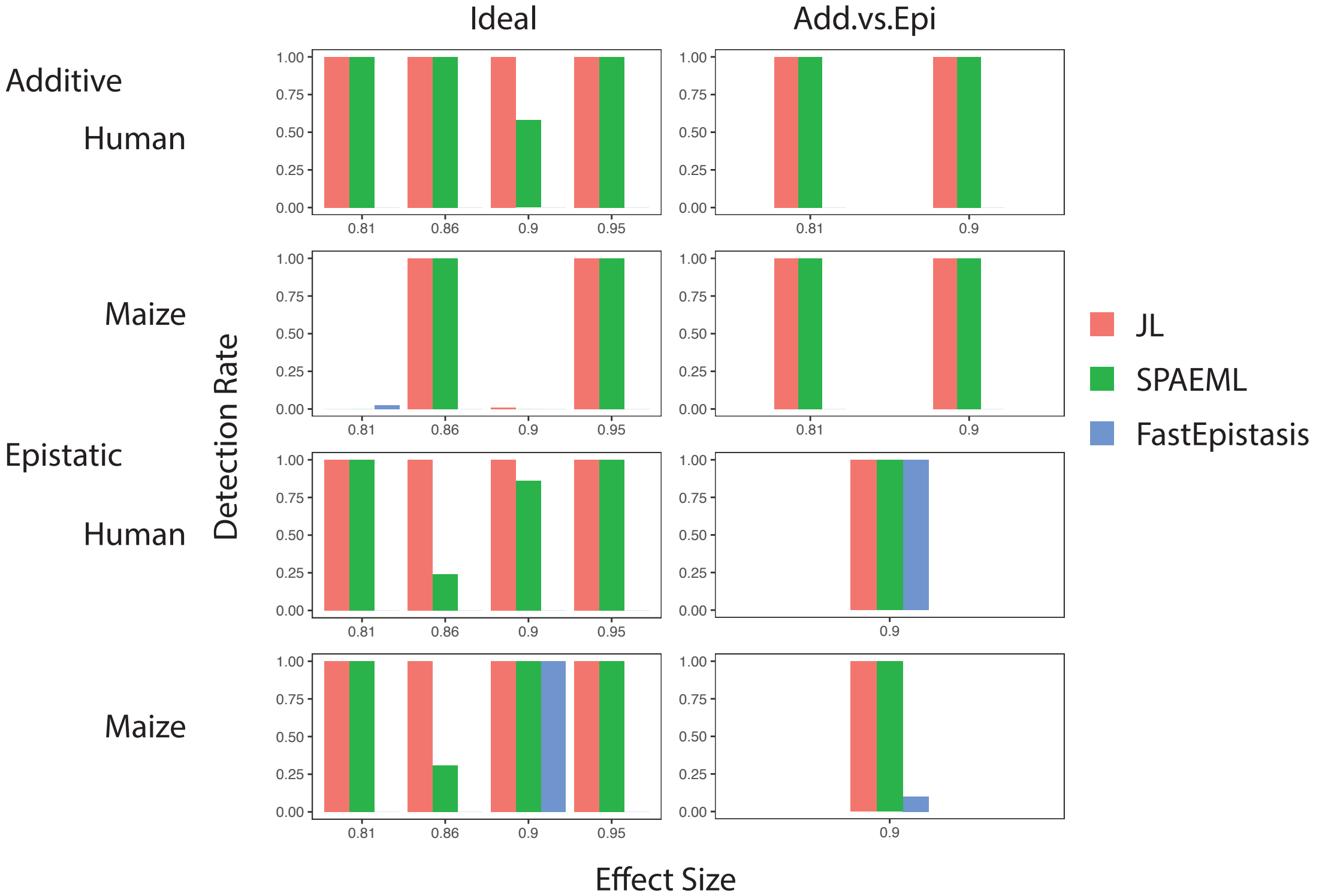

Supplement: Supplementary file 5 — Supplementary Figure 4 [file 41437_2018_162_MOESM5_ESM.pdf]

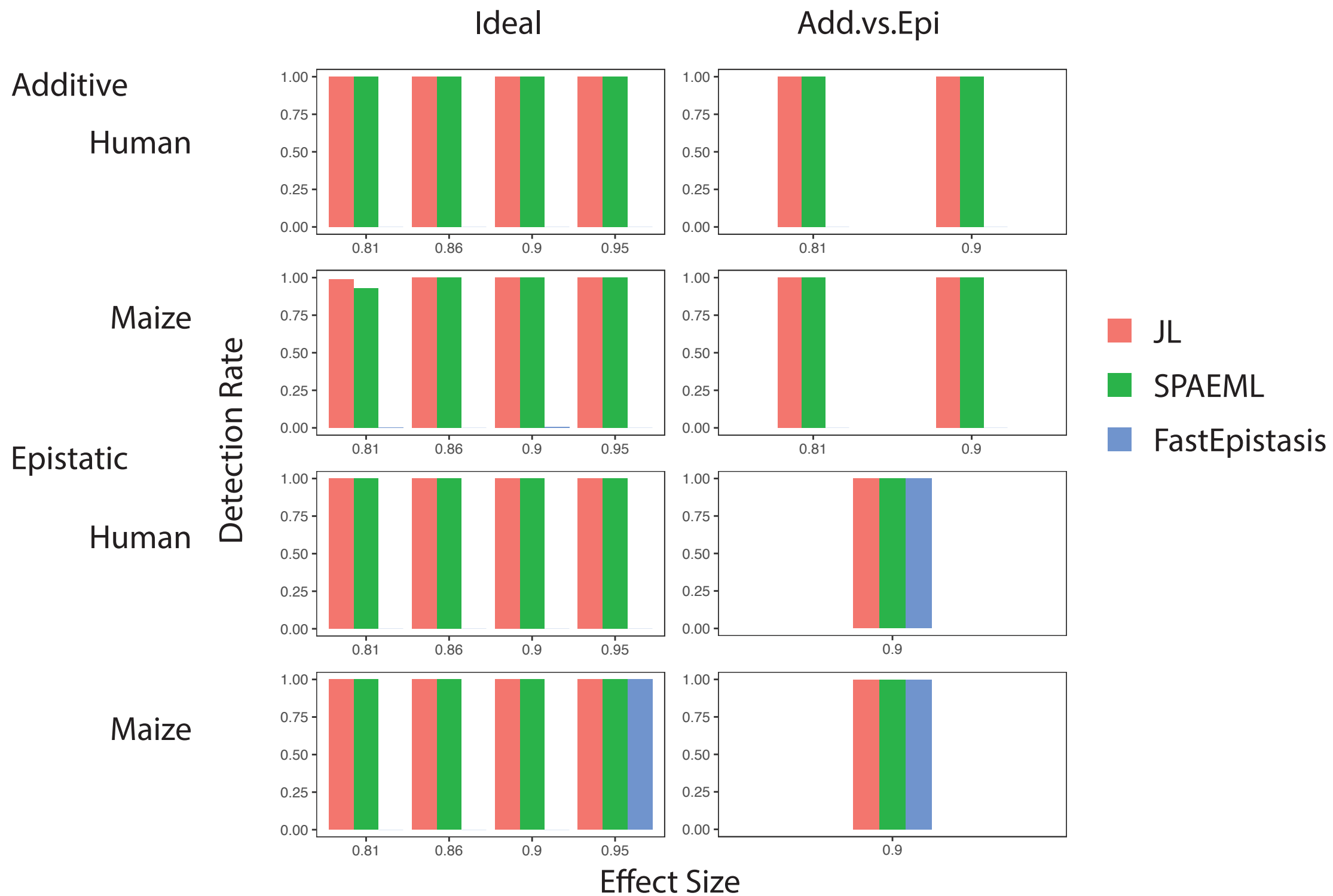

Supplement: Supplementary file 6 — Supplementary Figure 5 [file 41437_2018_162_MOESM6_ESM.pdf]

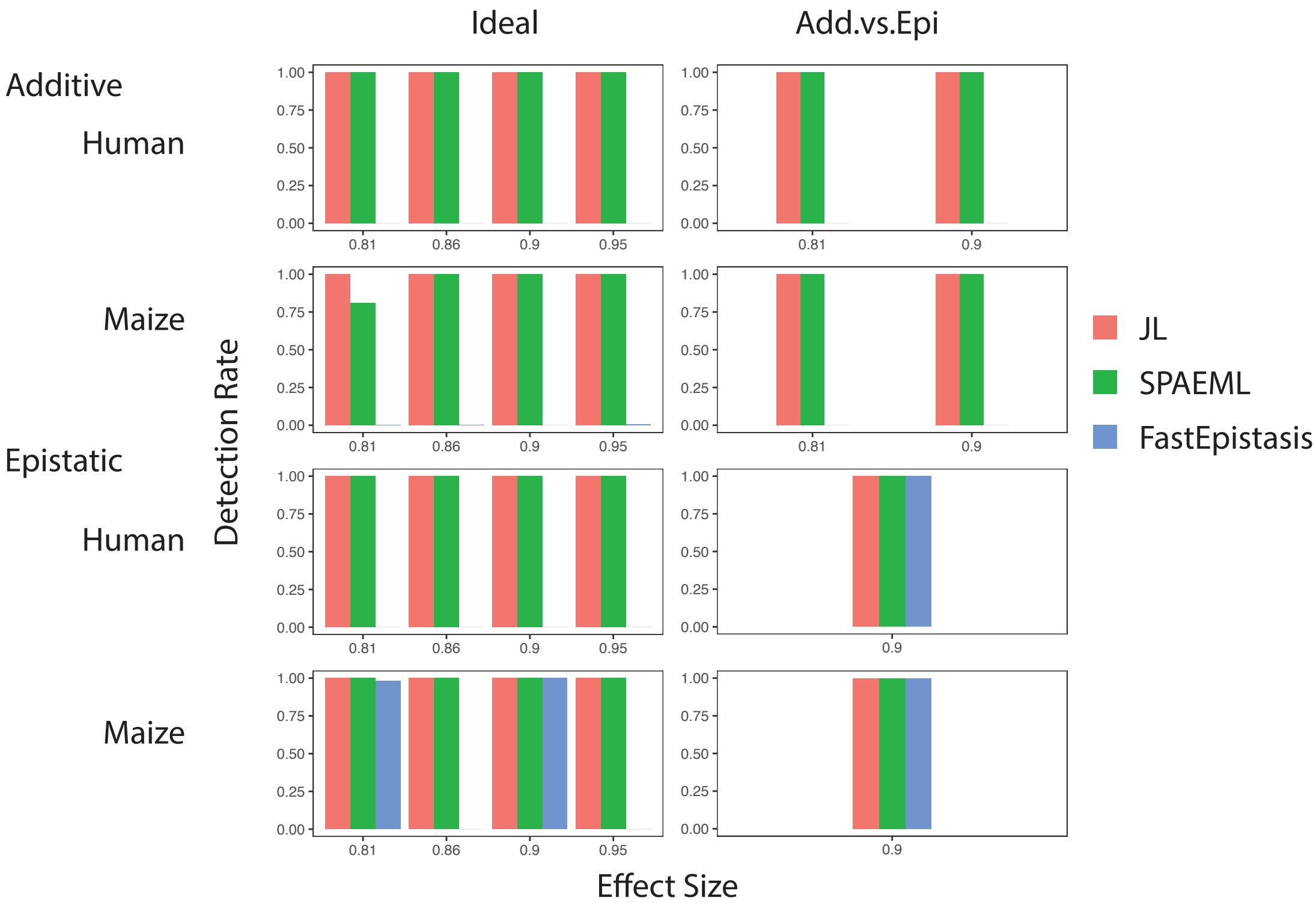

Supplement: Supplementary file 7 — Supplementary Figure 6 [file 41437_2018_162_MOESM7_ESM.pdf]

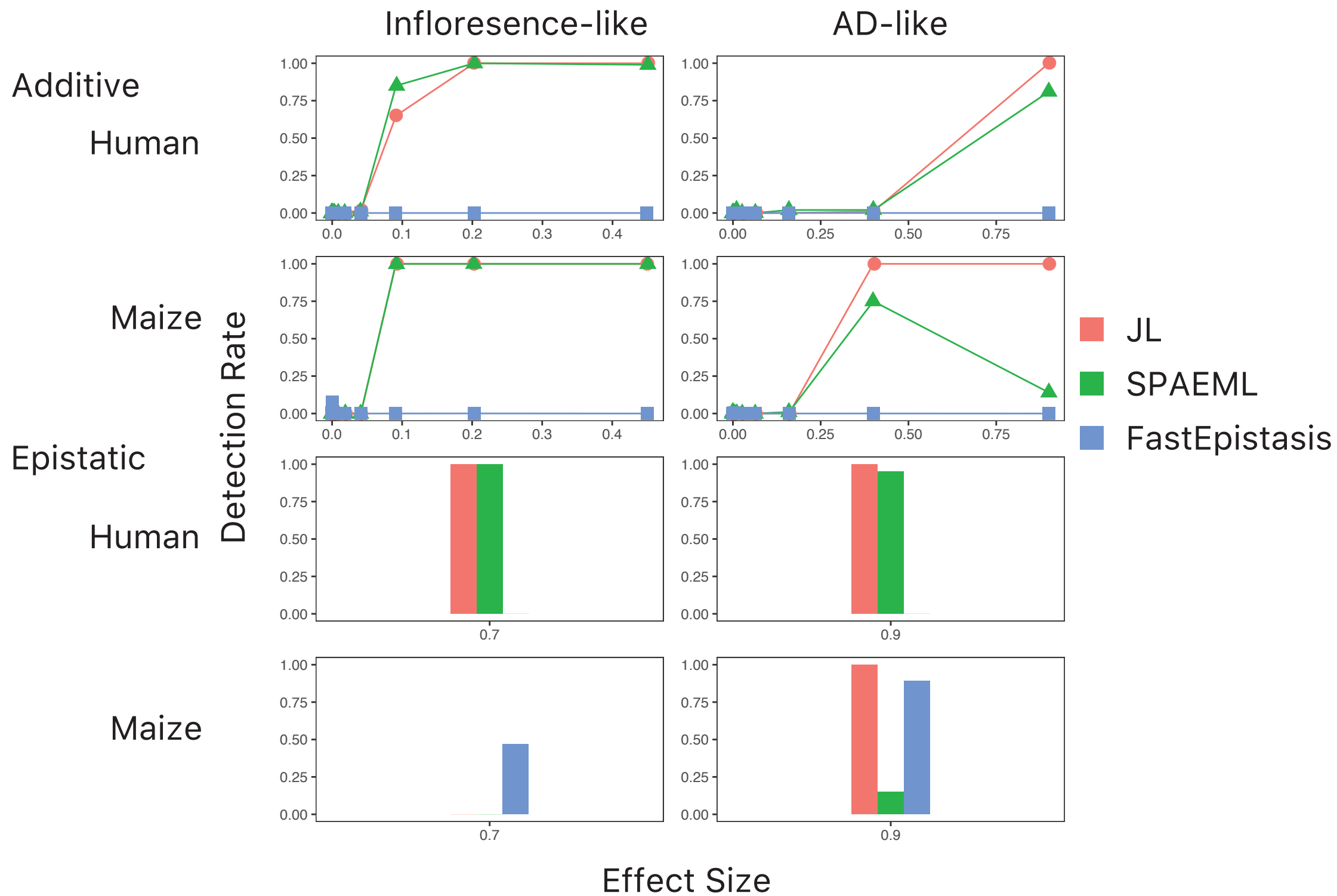

Supplement: Supplementary file 8 — Supplementary Figure 7 [file 41437_2018_162_MOESM8_ESM.pdf]

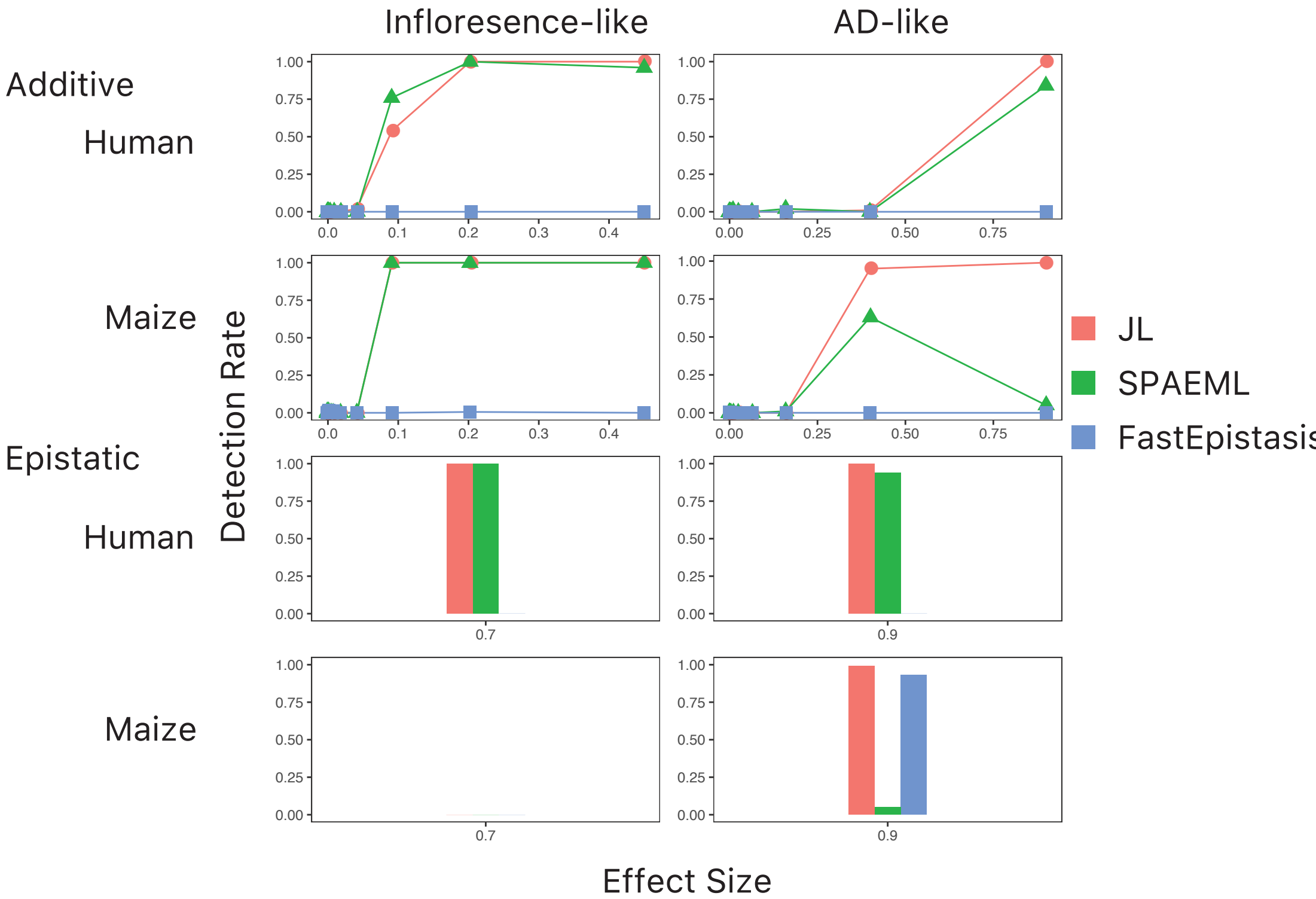

Supplement: Supplementary file 9 — Supplementary Figure 8 [file 41437_2018_162_MOESM9_ESM.pdf]

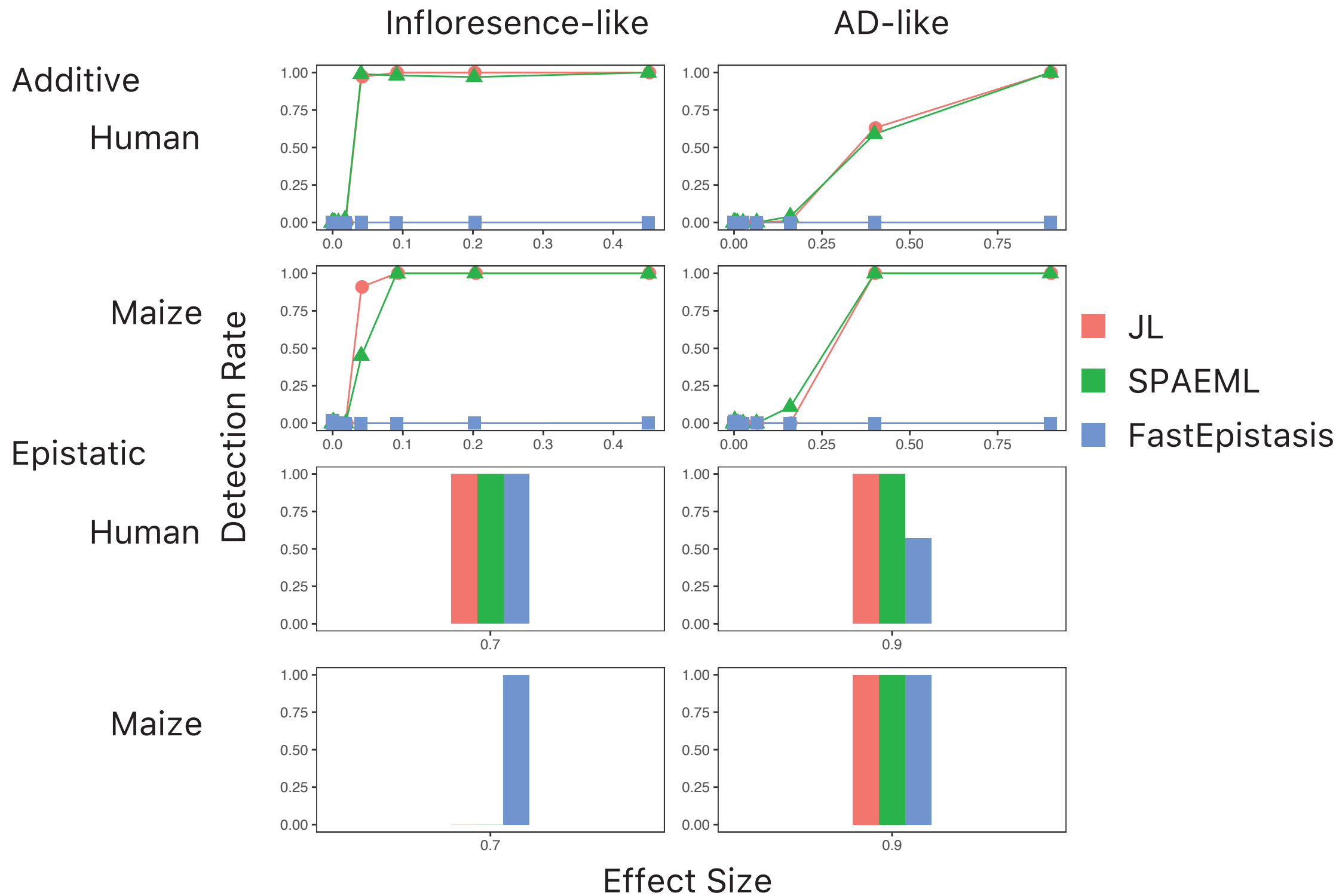

Supplement: Supplementary file 10 — Supplementary Figure 9 [file 41437_2018_162_MOESM10_ESM.pdf]

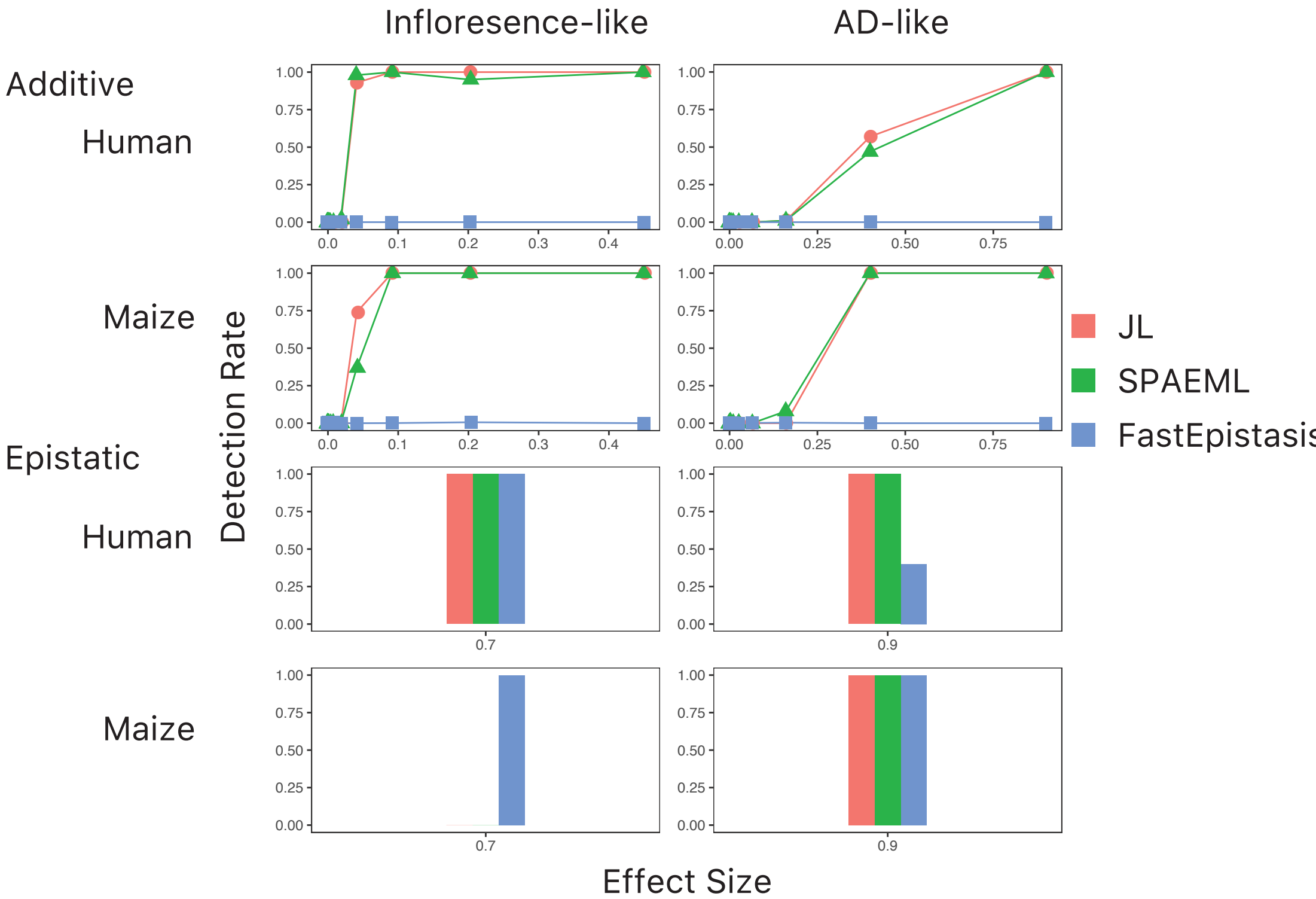

Supplement: Supplementary file 11 — Supplementary Figure 10 [file 41437_2018_162_MOESM11_ESM.pdf]

## Additive

# Human

# Maize

# Epistatic

# Human

# Maize

## Inflorescence-like

## AD-like

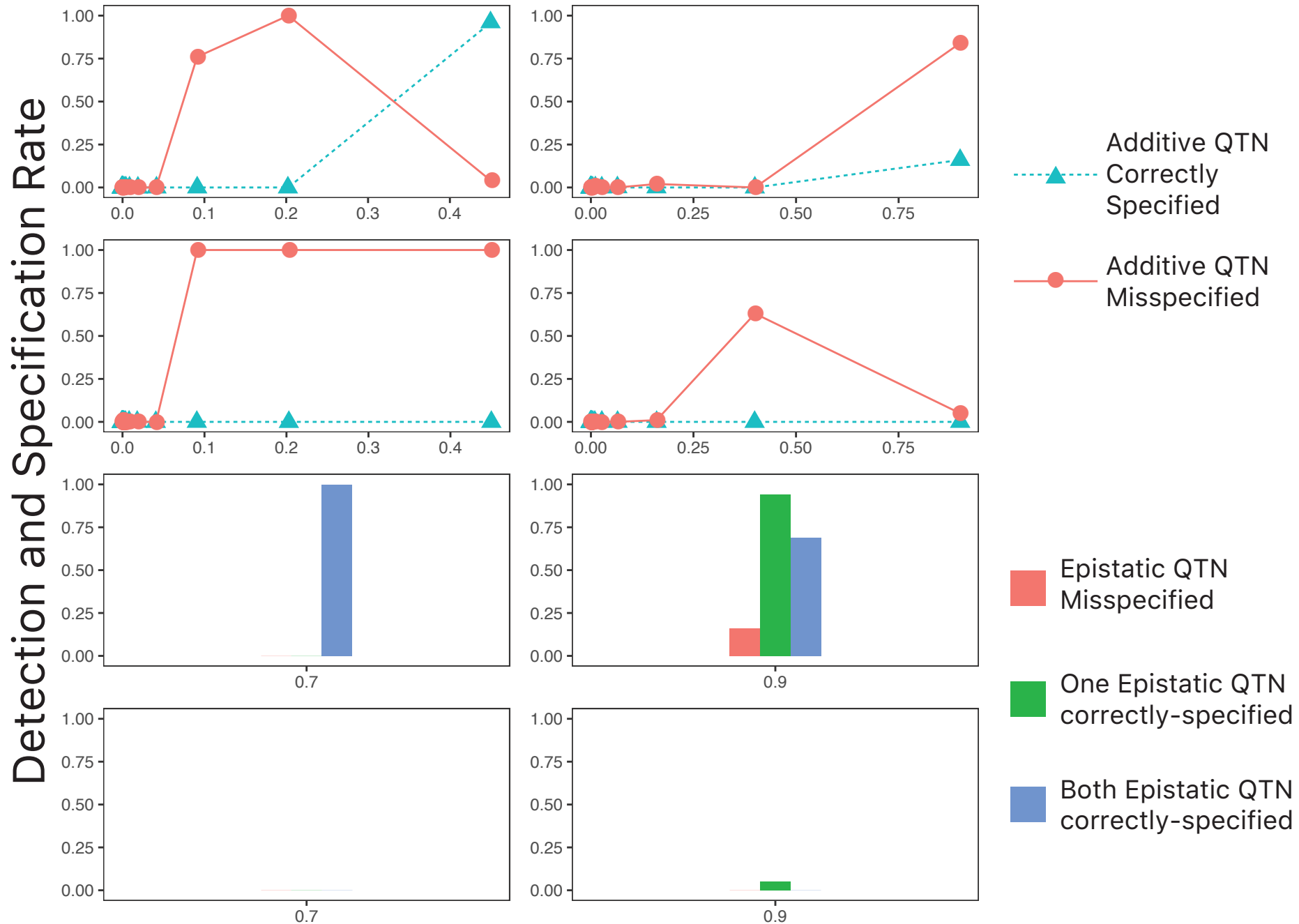

Supplement: Supplementary file 17 — Supplementary Figure 16 [file 41437_2018_162_MOESM17_ESM.pdf]
