# Supplementary material for: An assessment of true and false positive detection rates of stepwise epistatic model selection as a function of sample size and number of markers
Source: Heredity (Edinb). 2018 Nov 15;122(5):660–71. doi: 10.1038/s41437-018-0162-2 (PMC6462028; doi:10.1038/s41437-018-0162-2)
Supplement: Supplementary file 14 — Supplementary Figure 13 [file 41437_2018_162_MOESM14_ESM.pdf]

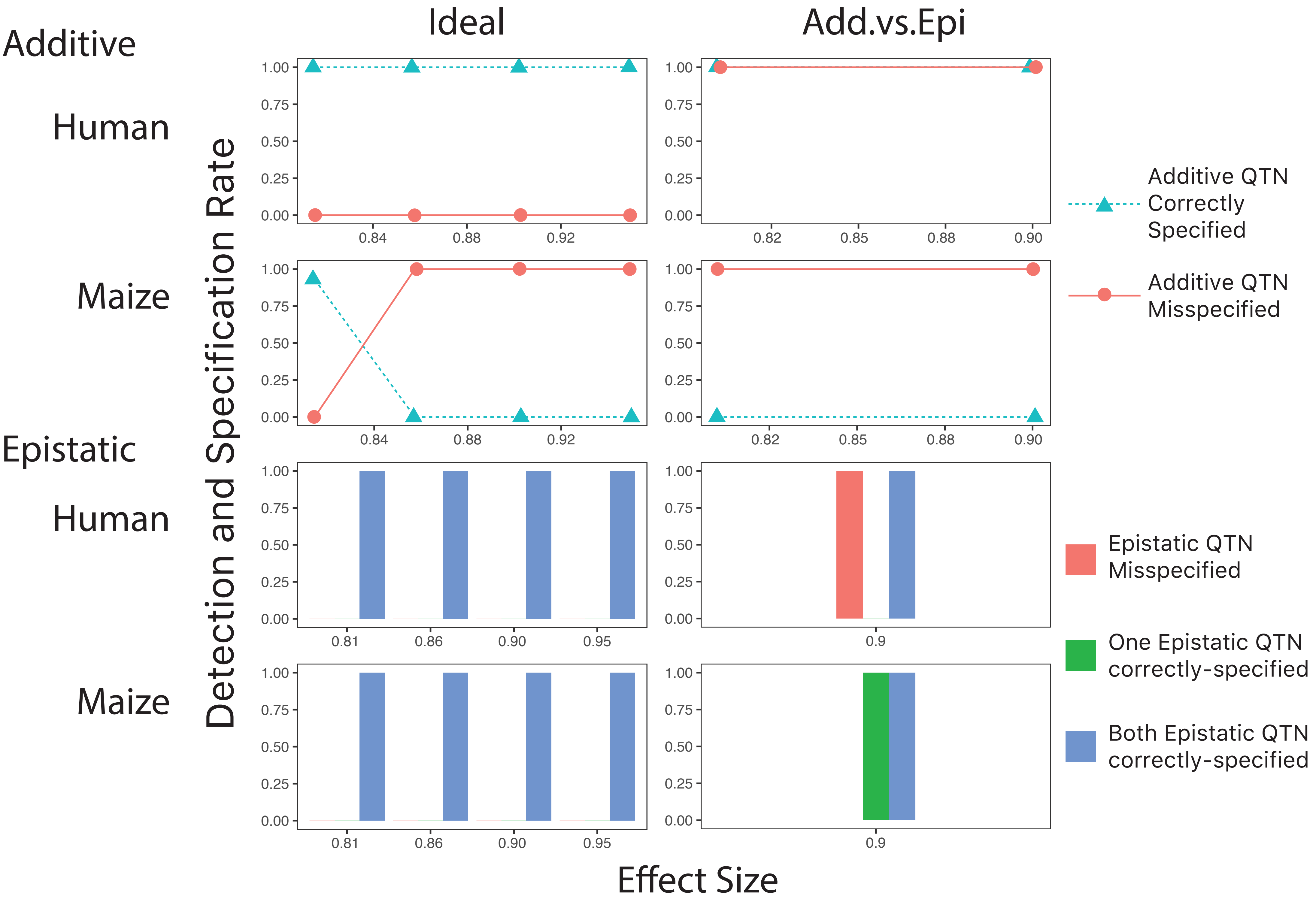

Ideal
Add.vs.Epi
Additive
Human
Maize
Epistatic
Human
Maize
Detection and Specification Rate
Effect Size
1.00
0.75
0.50
0.25
0.00
0.84
0.88
0.92
0.82
0.85
0.88
0.90
0.81
0.86
0.90
0.95
0.9
Additive QTN Correctly Specified
Additive QTN Misspecified
Epistatic QTN Misspecified
One Epistatic QTN correctly-specified
Both Epistatic QTN correctly-specified
