# Supplementary material for: An assessment of true and false positive detection rates of stepwise epistatic model selection as a function of sample size and number of markers
Source: Heredity (Edinb). 2018 Nov 15;122(5):660–71. doi: 10.1038/s41437-018-0162-2 (PMC6462028; doi:10.1038/s41437-018-0162-2)
Supplement: Supplementary file 18 — Supplementary Figure 17 [file 41437_2018_162_MOESM18_ESM.pdf]

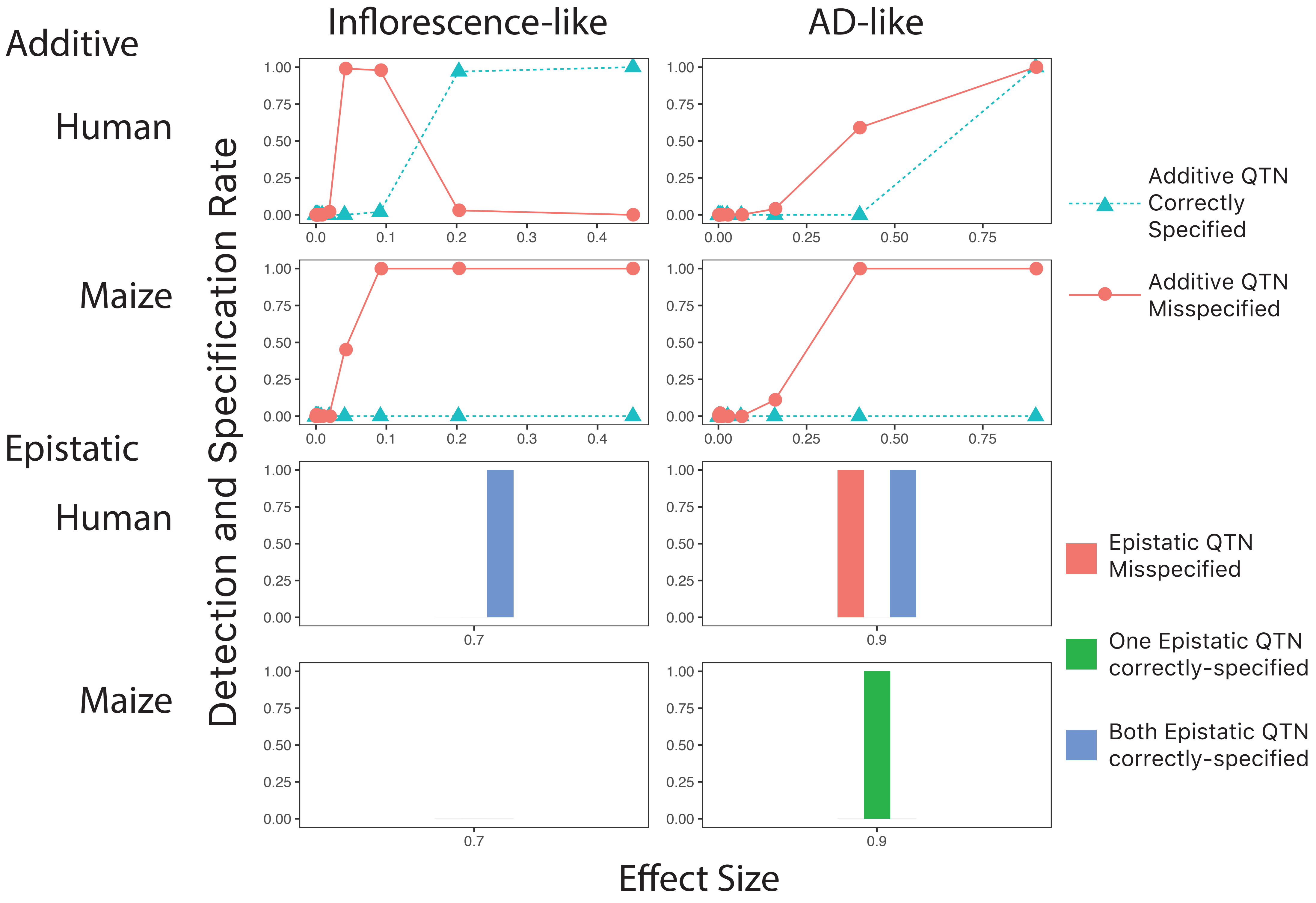

Inflorescence-like
AD-like
Additive
Human
Maize
Epistatic
Human
Maize
Detection and Specification Rate
Effect Size
Additive QTN Correctly Specified
Additive QTN Misspecified
Epistatic QTN Misspecified
One Epistatic QTN correctly-specified
Both Epistatic QTN correctly-specified
